# Targeting the Tumor Microenvironment with Fluorescence-Activatable Bispecific Endoglin/Fibroblast Activation Protein Targeting Liposomes

**DOI:** 10.3390/pharmaceutics12040370

**Published:** 2020-04-17

**Authors:** Felista L. Tansi, Ronny Rüger, Ansgar M. Kollmeier, Markus Rabenhold, Frank Steiniger, Roland E. Kontermann, Ulf K. Teichgräber, Alfred Fahr, Ingrid Hilger

**Affiliations:** 1 Department of Experimental Radiology, Institute of Diagnostic and Interventional Radiology, Jena University Hospital-Friedrich Schiller University Jena, Am Klinikum 1, 07747 Jena, Germany; ansgar.kollmeier@gmx.de (A.M.K.); ulf.teichgraeber@med.uni-jena.de (U.K.T.); 2 Department of Pharmaceutical Technology, Friedrich-Schiller-University Jena, Lessingstrasse 8, 07743 Jena, Germanyalfred.fahr@uni-jena.de (A.F.); 3 Center for Electron Microscopy, Jena University Hospital-Friedrich Schiller University Jena, Ziegelmuehlenweg 1, 07743 Jena, Germany; frank.steiniger@med.uni-jena.de; 4 Institute of Cell Biology and Immunology, University Stuttgart, Allmandring 31, 70569 Stuttgart, Germany; roland.kontermann@izi.uni-stuttgart.de

**Keywords:** liposomes, molecular targeting, fluorescence quenching, optical imaging, tumor microenvironment, tumor heterogeneity

## Abstract

Liposomes are biocompatible nanocarriers with promising features for targeted delivery of contrast agents and drugs into the tumor microenvironment, for imaging and therapy purposes. Liposome-based simultaneous targeting of tumor associated fibroblast and the vasculature is promising, but the heterogeneity of tumors entails a thorough validation of suitable markers for targeted delivery. Thus, we elucidated the potential of bispecific liposomes targeting the fibroblast activation protein (FAP) on tumor stromal fibroblasts, together with endoglin which is overexpressed on tumor neovascular cells and some neoplastic cells. Fluorescence-quenched liposomes were prepared by hydrating a lipid film with a high concentration of the self-quenching near-infrared fluorescent dye, DY-676-COOH, to enable fluorescence detection exclusively upon liposomal degradation and subsequent activation. A non-quenched green fluorescent phospholipid was embedded in the liposomal surface to fluorescence-track intact liposomes. FAP- and murine endoglin-specific single chain antibody fragments were coupled to the liposomal surface, and the liposomal potentials validated in tumor cells and mice models. The bispecific liposomes revealed strong fluorescence quenching, activatability, and selectivity for target cells and delivered the encapsulated dye selectively into tumor vessels and tumor associated fibroblasts in xenografted mice models and enabled their fluorescence imaging. Furthermore, detection of swollen lymph nodes during intra-operative simulations was possible. Thus, the bispecific liposomes have potentials for targeted delivery into the tumor microenvironment and for image-guided surgery.

## 1. Introduction

Liposomes are highly biocompatible organic nanovesicles with a high drug payload capacity [1] and flexibility for modifications, whereby different substances including genes, proteins, therapeutic drugs and contrast agents are encapsulated in the aqueous interior or embedded in the lipid bilayer [1,2,3]. Thus, liposomes are eminent for biomedical research on disease pathogenesis, drug delivery and therapy monitoring purposes. The clinical use of non-targeted PEGylated liposomes for the delivery of chemotherapeutics [4] greatly relies on the enhanced permeability and retention (EPR)-effect and the level of phagocytic tumor associated macrophages (TAMs) available in the tumor stroma [5]. Although this passive liposomal delivery has greatly increased the efficacy of tumor treatment as compared to the free chemotherapeutic drugs, they still have limitations. Thus, the low therapeutic efficacy seen in some patients receiving liposomal chemotherapy can be ascribed to low availability of TAMs or poor leakiness of the tumor vasculature. In some cases, the depletion of TAMs could impact therapy negatively, since high levels of TAMs have revealed better prognosis and survival rates than in patients with low TAM numbers [6,7,8]. Passive drug delivery based on the EPR-effect is also thought to enhance the development of multidrug resistance (MDR) in many patients [9,10]. Thus, the high heterogeneity seen between patients with the same cancer types raises the need for unique biocompatible vehicles that can deliver drugs and contrast agents directly into distinct target tumor cells and the tumor stroma for the future imaging and therapy of various tumors. So far, many peptide, antibody and protein based molecular targeting agents [11,12] as well as monospecific targeted liposomes [5,13] have been developed, but are either limited by rapid clearance and poor tumor penetration [11] or can address only one target marker at a time. Moreover, peptide- and antibody-drug conjugates have limited cargo-payload capacities. Considering the high tumor heterogeneity seen in cancers, the availability of the target marker is a limiting factor in molecular targeting. Hence, the use of liposomes that are targeted towards two or more tumor markers would raise the chance of accessing at least one of them by the bispecific liposome. For example, the modification of doxorubicin-bearing liposomes with ligands targeting the tumor markers, CD19 and CD20 improved the selectivity of delivery and overall therapeutic response of lymphoma models [14]. However, CD19 and CD20 are not universal targets expressed in many cancer types, and as such cannot be implemented in a broad spectrum of patients. We therefore hypothesized that endowing liposomes with ligands that are selective for more universal tumor markers found in a diverse spectrum of tumor types could be beneficial for many patients.

Many cell surface receptors are elevated on tumor cells and the tumor stroma, and hence represent unique markers for targeted drug delivery. The fibroblast activation protein (FAP) for example, is exclusively overexpressed on the stromal fibroblasts of healing wounds [15], rheumatoid arthritis [16] and on 90% of tumor associated fibroblasts in a broad spectrum of epithelial cancers [17,18], making it a universal tumor marker. FAP is a type II transmembrane sialoglycoprotein with known exo-/endopeptidase activity for collagens, and for biological peptides and proteins that carry an N-terminal alanine or proline on the penultimate position. Elevated levels of activated tumor myofibroblasts are accompanied by high levels of FAP, and correlate with an invasive phenotype [19]. Thus, FAP-based targeting holds promising potentials for the management of epithelial cancers [17]. Regrettably, a humanized monoclonal antibody considered for the FAP-targeted therapy, failed a phase II clinical trial of metastatic colorectal cancers [20]. This is not very surprising, since many of the FAP enzymatic products are signaling molecules involved in immune suppression [21] and FAP also has unknown tumor growth modulating functions that are irrespective of its enzymatic activity [22]. Thus, different approaches need to be implemented in targeting FAP to deplete the stromal fibroblasts in cancer. Considering the high heterogeneity seen in cancers, and the fact that FAP enhances tumor growth and formation of metastases [22,23,24] by modulating tumor vessel formation and migration of myofibroblasts into tumors [25], the consideration of other stromal markers for bispecific tumor targeting alongside FAP, is vital. Interestingly, the integral membrane protein endoglin (CD105) also conveys characteristic features of a potent tumor stromal marker for cancer imaging and therapy. Endoglin is an essential part of the transforming growth factor (TGF)-beta receptor complex [26], which is involved in the formation of new blood vessels in many diseases, including cancers [27,28]. It is expressed only at low levels on the vasculature of normal tissues, but elevated on some neoplastic cells, the neovasculatures of many cancers, and other pathological conditions, such as preeclampsia and hereditary hemorrhagic telangiectasia [29], and hence is a universal target for diagnosis and anti-angiogenic therapy [27,30,31,32].

Considering the beneficial features of FAP and endoglin as target markers and also previous works where we demonstrated tumor imaging based on the use of activatable mono-specific liposomes, we therefore sought to elucidate the feasibility of targeting FAP and endoglin simultaneously, with activatable bispecific liposomes. In this respect, we prepared bispecific liposomes with 3 main peculiarities, for the simultaneous targeting of FAP and endoglin on the tumor stroma. Firstly, the liposomes were encapsulated with high concentrations of the near-infrared fluorescent (NIRF) dye, DY-676-COOH, which is intrinsically quenched at high concentrations. In previous studies, we demonstrated that activatable, non-targeted, PEGylated liposomes (Lip-Q) prepared in the same way were fluorescence quenched and could fluoresce exclusively after their cellular uptake and activation by phagocytic cells [33,34]. Moreover, we showed that the conjugation of FAP-specific single chain antibody fragments (scFv) to the quenched liposomes reduced their uptake by phagocytic cells, and enhanced their affinity for FAP-expressing cells, thereby enabling fluorescence imaging of FAP expressing xenografts in mice [35]. The second peculiarity of the bispecific liposomes was the presence of a non-quenched green fluorescent phospholipid embedded in their lipid bilayer. Since the green-fluorescent phospholipid is always fluorescent even when the liposomes are intact and NIRF-quenched, the intact liposomes within cells/tumors can be traced prior to their degradation and activation of the encapsulated NIRF dye. The third key peculiarity of the bispecific liposomes was the presence of 5% PEGylation onto which scFv directed towards human FAP and murine endoglin (FAP’scFv and mEdg’scFv) were coupled. The tumor stroma including the tumor blood vessels and fibroblasts originate from the host (mouse) when human tumor cells are xenografted as done herein. Since human and mouse FAP protein have a high amino acid sequence homology and antibody cross-reactivity [36], the human FAP’scFv on the liposomal surface would react with murine fibroblasts on the tumor stroma of xenografted human cancer models in mice. In contrast, human and mouse endoglin proteins have low amino acid sequence homology and no antibody cross-reactivity [37]. Thus, to correctly elucidate the vascular targeting potential of the bispecific liposomes in mice models, murine endoglin scFv was used. Thus, we envisioned that the bispecific FAP and endoglin targeting liposomes (termed Bi-FAP/mEnd-IL, and abbreviated Bis-IL in the Figures) should be able to accumulate in the tumors based on distinct specificities to the murine FAP-expressing tumor associated fibroblasts, murine endoglin-expressing tumor vascular endothelial cells, and for tumor models that express the human FAP protein, also the tumor cells. We validated this concept in xenografted mice models based on human fibrosarcoma cells that stably expressed high levels of human FAP protein, and also a human breast carcinoma model that lack FAP expression. Respective mono-specific FAP- (FAP-IL) and murine endoglin (mEnd-IL) targeting and non-targeted quenched liposomes (LipQ) were used for comparison.

The results demonstrate highly fluorescence-quenched Bi-FAP/mEnd-IL and their selective binding to FAP and murine endoglin on cultured cells and in vivo in tumor models in mice. Compared to the monospecific liposomes, the bispecific liposomes do not increase the overall payload delivered into the tumor at comparative time points post application, owing to a target-ligand based alteration in the tumor accumulation kinetics, but precisely deliver the cargoes into both the tumor stromal fibroblasts and tumor neovascular endothelia, as could be pin-pointed by confocal microscopy. Furthermore, the bispecific liposomes enhanced the live NIRF detection of suspicious swollen lymph nodes in intra-operative simulations. Thus, we are convinced that the Bi-FAP/mEnd-IL, similar to the monospecific liposome formulations represents a potent tool for the detection of tumor margins and metastatic tissues during surgical interventions. Moreover, it over-scores the monospecific liposomal formulations in the capacity to deliver cargos into the stromal fibroblasts and tumor vascular cells simultaneously. Hence, modification of the Bi-FAP/mEnd-IL to bear human endoglin targeting scFv and the encapsulation of therapeutic substances such as nucleotides, chemotherapeutics and other insoluble drugs merit consideration for the future management of a broad spectrum of human solid cancers.

## 2. Materials and Methods

### 2.1. Lipids and Materials Used for Liposome Preparation

All phospholipids, including egg phosphatidylcholine (EPC), cholesterol (chol), 1,2-distearoyl-sn-glycero-3-phosphoethanolamine-N-[methoxy (polyethylene glycol)-2000] (ammonium salt) (mPEG2000-DSPE), 1,2-distearoyl-sn-glycero-3-phosphoethanolamine-N-[maleimide(polyethylene glycol)-3400] (ammonium salt) (MalPEG_3400_-DSPE), and 1,2-dioleoyl-sn-glycero-3-phosphoethanolamine-N-(7-nitro-2-1,3-benzoxadiazol-4-yl) (ammonium salt) (NBD-DOPE) were commercially acquired from Lipoid GmbH (Ludwigshafen, Germany) and Avanti Polar Lipids (Alabama, USA). The detergent Tris (hydroxymethyl)-aminomethane (Tris) and 4-(1,1,3,3-Tetramethylbutyl)phenyl-polyethylene glycol (Triton-X100) was purchased from Sigma (Taufkirchen, Germany), and the near infrared fluorescent (NIRF) dye, DY-676-COOH was acquired from DYOMICs GmbH (Jena, Germany).

### 2.2. Preparation and Physicochemical Characterization of Quenched Liposomes

The film hydration and extrusion method was implemented to prepare fluorescence-quenched liposomes. Hereby, a lipid film of the composition EPC:Chol:mPEG_2000_-DSPE at a molar ratio of 6.5:3:0.5 and 0.3 mol% of the lipophilic marker NBD-DOPE were hydrated with a 4.3–5 mM concentration of the NIRF dye, DY-676-COOH (excitation/emission: 674 nm/699 nm) in 10 mM Tris pH 7.4. After seven cycles of freezing and thawing, the liposomes were extruded, purified by gel-filtration, and characterized as reported earlier [5,33,35]. Due to their encapsulation within the aqueous interior of the liposomes the highly concentrated DY-676-COOH undergoes intrinsic fluorescence-quenching. Therefore, the green fluorescent phospholipid NBD-DOPE (excitation/emission: 480 nm/530 nm) which does not undergo self-quenching was embedded in the lipid bilayer to enable detection and demarcation of intact liposomes in cells prior to their degradation, release and activation of the NIRF dye.

### 2.3. Preparation and Conjugation of FAP and Murine Endoglin Antibody Fragments to Preformed Quenched Liposomes

Single chain antibody fragments (scFv) specific for the human FAP protein were purified by metal affinity chromatography following their periplasmic preparation from *Escherichia coli* (E. coli) as described previously [38]. Likewise, plasmid DNA encoding the murine endoglin single chain antibody fragment acquired by phage display (termed scFv-mE12) as reported previously [39] were transfected into human embryonal kidney (HEK293T) cells using Lipofectamine^TM^ 2000 (Invitrogen, Carlsbad, CA, USA). Stable clones were selected with 500 µg/mL Zeocin (Invitrogen), expanded in RPMI medium containing 5% fetal calf serum (FCS), both from Gibco^®^, (Paisley, Scotland) then further cultured in Opti-MEM^®^ medium which induces the expression and secretion of the scFv proteins. The murine endoglin scFv was purified from the culture supernatant by immobilized metal affinity chromatography (IMAC) according to previous reports [40]. The purified FAP and murine endoglin scFv were coupled to MalPEG_2000_-DSPE and MalPEG_3400_-DSPE micelles, respectively for 60 min at room temperature, validated by polyacrylamide gel electrophoresis before and after conjugation to micelles in order to deduce the coupling efficiency and purity.

Thereafter, 0.3 mol% murine endoglin- or 0.1 mol% human FAP-scFv-conjugated micelles (MalPEG_3400_-scFv and MalPEG_2000_-scFv) were post-inserted into preformed quenched liposomes with at 50 °C for 60 min as described earlier [5]. Both the FAP and murine endoglin scFv conjugated micelles were post-inserted simultaneously to acquire the bispecific fluorescence quenched liposomes. The amount of scFv molecules inserted in the liposomes could be estimated with respect to the micellar lipid titration protocols (Supplementary data S1) and also previous reports [41,42]. The resulting FAP, murine endoglin and bispecific targeted, quenched liposomes were termed FAP-IL, mEnd-IL and Bi-FAP/mEnd-IL, respectively. Murine and human FAP proteins share a very high amino acid sequence homology. Thus, the human scFv antibody cross reacts with murine FAP protein and should bind to murine FAP positive tumor stromal cells in xenografted human cancer models in mice. In contrary, murine and human endoglin share very low amino acid sequence homology, hence no antibody cross reactivity of the mEnd-IL to human endoglin on the endoglin positive human cancer cells is expected. The quenched liposomes (LipQ) used in this study as control were post-inserted with empty micelles generated after cysteine reduction of MalPEG_2000_-DSPE to achieve comparable properties with the targeted liposomes. Physicochemical characterization by dynamic light scattering, spectrometry and electron microscopy were implemented to estimate dye content, lipid concentration, size, zeta potential and morphology of the liposomal vesicles as described in detail earlier [5].

### 2.4. Determination of the Liposomal Fluorescence Quenching and Activation Property

Fluorescence-quenching of the liposomes which is characterized by a blue-shift in the absorption wavelength and a re-shift towards red wavelengths were validated in vitro. For this, the absorption (Ultrospec 4000 photometer) and fluorescence emission (Jasco FP6200 spectrofluorometer, Jasco Gross-Umstadt, Germany) of the liposomes (100 nmol (final lipids) in 100 µL 10 mM Tris pH 7.4) were measured before and after triggering liposomal membrane damage by freezing at −80 °C as described previously [33].

### 2.5. Cell Lines, Media and Culture Conditions Implemented

The human fibrosarcoma cell line, HT-1080 which overexpresses endogenous human endoglin was purchased from Cell Lines Services (CLS, Heidelberg, Germany). MDA-MB231, a human breast carcinoma cell line with low levels of endogenous human endoglin expression was also purchased from Cell Lines Services. The murine melanoma cell line B16F10 which expresses low levels of endogenous murine endoglin, was stably transfected with a vector DNA bearing murine endoglin gene in previous work [39] to get the high murine endoglin expressing cell line, B16F10mCD105 used here. The MDA-MB231 cells were cultured in Dulbecco’s modified Eagle’s medium (DMEM) supplemented with 10% (*v*/*v*) fetal calf serum (FCS) and 1% Hepes (Gibco^®^, Paisley, Scotland), whereas the HT-1080 cell line, its stable FAP expressing counterpart HT1080-hFAP, and the B16F10mCD105 cells were cultured in RPMI medium supplemented with 5% (*v*/*v*) FCS (Gibco^®^). Standard culture conditions (37 °C, 5% CO_2_ and 95% humidified atmosphere) were applied for all the cell lines.

### 2.6. Validation of the Liposomal Binding and Uptake Selectivity in Cultured Target Cells

The ability of the different liposomal formulations to bind and be taken up selectively by target cells expressing the FAP protein or the murine endoglin protein was validated quantitatively by fluorescence-activated cell sorting, (FACS) and NIRF imaging, and also qualitatively by confocal laser scanning microscopy as reported previously [5]. Cells cultured under standard conditions were washed trypsinized and counted and then subjected to further use as follows: for quantitative analysis by FACS, 5 × 10^5^ cells (HT1080-FAP or B16F10mCD105) were dispensed in PBS containing 490 µMol (final lipids) of the respective targeting and control liposomes either alone or together with 10 µg of the murine Endoglin scFv (mEdg’scFv) and human FAP’scFv as a competing blocker of binding. The cells were incubated for 2 h at 4 °C then washed and analyzed on an EPICS XL-MCL flow cytometer (Beckmann Coulter GmbH, Krefeld, Germany).

Furthermore, 2 × 10^6^ cells (HT1080-FAP, MDA-MB231 or B16F10mCD105) were seeded in small tissue culture flasks (or quantitative analysis of the internalization and liposomal activation by NIRF imaging), whereas 30,000 cells (HT1080-FAP, HT1080, MDA-MB231 or B16F10mCD105) were seeded on poly-L-lysine coated 8-well chamber culture slides (BD Biosciences) for subsequent microscopy. The cells in flasks and culture slides were further cultured overnight at standard conditions. Thereafter, 200 nmol (final lipids) of the respective liposomes were added and subsequently cultured for 8 h at 37 °C or for 2 h–24 h (time course studies) under standard culture conditions. The cells in flasks were washed, scraped and transferred to small reagent tubes in PBS and then pelleted by centrifugation at 200 g. The cell pellets were subsequently imaged in the Maestro^TM^ fluorescence imaging system using the parameters described previously [5]. Cells grown on chamber slides were washed and simultaneously mounted and nuclei-stained by diluting Hoechst-33258 (Applichem, Darmstadt, Germany) in Permafluor (ThermoFischer Scientific, Dreieich, Germany) mounting solution. The cells were subsequently covered with glass coverslips and subjected to confocal laser scanning microscopy on an LSM510 (Zeiss, Jena, Germany) as reported earlier [16,18]. The nuclei were visualized with a 405 nm laser diode and a 420–480 nm band pass filter, whereas NBD-DOPE fluorescence was excited at 488 nm and detected at 530 nm. Likewise, the DY-676-COOH was excited with a 633 nm Argon laser and images captured with a 650 nm long pass filter. All acquisitions were done at a 63× magnification.

### 2.7. Animals and Implantation

All trials conducted on animals were approved by the regional animal committee (Thueringer Landesamt fuer Lebensmittelsicherheit und Verbraucherschutz, Bad Langensalza, Germany) under the numbers 02-039/09 and 02-047/11, and in conformation with international guidelines on the ethical use of animals. Female immune deficient athymic nude mice (Hsd:Athymic Nude-Foxn1^nu^ nu/nu; Harlan Laboratories, the Netherlands) of age 10–18 weeks and weighing approximately 24 g were used for tumor studies. For biodistribution study in immune-competent mice, 8–12 weeks old male NMRI mice (Elevage Janvier, Le Genest Saint Isle, France) were used. The mice were housed under standard conditions with ad libitum mouse chow and water. 2–4 weeks prior to in vivo imaging, tumor models were induced by injecting 2.0 × 10^6^ cells dispensed in 120 µL cold Matrigel™ (BD Biosciences) subcutaneously into the lower back of the mice. The animals were anesthetized with 2% isoflurane throughout the procedure.

### 2.8. Determination of Tumor Volumes

The length, width and heights of tumors were measured with a digital caliper and used to calculate the corresponding tumor volumes in two dimensions according to Feldmann [43].

### 2.9. Whole Body NIRF Imaging of Mice, Excised Organs and Determination of Fluorescence Intensities

Approximately, 2–4 weeks after tumor implantation, mice bearing subcutaneous xenografts with diameters of the range 5–10 mm were subjected to tumor imaging. Mice were anesthetized with 2% isoflurane and intravenously injected with 20 µmol/kg body weight (final lipids) of the respective liposomes diluted in PBS (to 5 mL/kg body weight final volume). The non-targeted quenched LipQ served as control. Animals were imaged immediately after injection (time point, t = 0 h post injection (p.i.)). Further images were acquired every 2 h for 10 h and again at t = 24–32 h and at 48 h p.i. The Maestro^TM^ in vivo fluorescence imaging system (Cri-InTAS, Woburn USA) was used for acquisition, employing the red filter set (Excitation range 615–665 nm and emission >700 nm (cut-in filter)). Image evaluation was done by unmixing background auto-fluorescence acquired for native animals prior to probe injection, using the Maestro 3.0 software. The fluorescence intensities of tumors as compared to background (muscle) were derived by choosing regions of interest (ROIs) on the tumors and thigh regions of mice respectively. For liposomal biodistribution studies tumor free female athymic nude and male NMRI mice were intravenously injected with the liposomes and sacrificed 6 h post injection. Then their organs were excised and imaged similar to the whole animals. Semi-quantitative analyses of the tumor and organ fluorescence intensities were done by assigning a region of interest (ROI) to the desired region on each of the unmixed, intensity scaled (for exposure time, camera gain, binning and bit depth) tumors or organs as reported earlier [5]. The fluorescence intensities of the individual ROIs were deduced as average signal (scaled counts/s), which makes the analysis comparable with one other.

### 2.10. Euthanasia and Ex Vivo Determination of the Biodistribution of Liposomes

Mice were anesthetized with 2% isoflurane until reaction to touch was no longer detected, and then sacrificed with carbon dioxide until breathing stopped completely. The organs and tumors were excised, imaged and the fluorescence intensities semi-quantitatively deduced as described above.

### 2.11. Verification of the Localization of Liposome Signals in Freshly Isolated Tissues

Freshly excised tumor and liver and kidneys from mice injected with the respective liposomes were excised immediately subjected to fresh-tissue confocal microscopy as shown previously [44,45]. Hereby, the excised tissues of interest were quickly rinsed in sterile PBS and a small piece was smoothly cut and placed with the smooth surface lying on a glass coverslip of a Lab-Tek™ 4-well borosilicate cover-glass system (Thermo-Scientific, Dreieich, Germany), then subsequently imaged on a confocal laser scanning microscope (Zeiss). In this constellation, the tissue auto-fluorescence (blue to green fluorescence) of the non-processed fresh tissues grants visualization of the tissue morphology. Thanks to the liposomal DY-676-COOH fluorescence absorption and emission maxima beyond the auto-fluorescence range (e.g., liposomal DY-676-COOH: abs/em. 674/699 nm) the localization of the liposomes can be visualized within the organs. The image acquisition was done at a 20× magnification with similar excitation and emission parameters used for the cellular uptake experiments described above.

### 2.12. Statistical Evaluation of Significance

Student’s *t*-test and 2-way Anova tests were used to verify the level of significance, when normality and equal variance tests were passed. Otherwise, the Mann-Whitney-Rank sum test was used. All experiments were done at least three times. Except otherwise indicated, five or more mice/group were used for in vivo studies. Differences resulting in *p* < 0.05 were regarded as being significant.

## 3. Results

### 3.1. Preparation and Physicochemical Characterization of Activatable Liposomes

**Figure 1:**
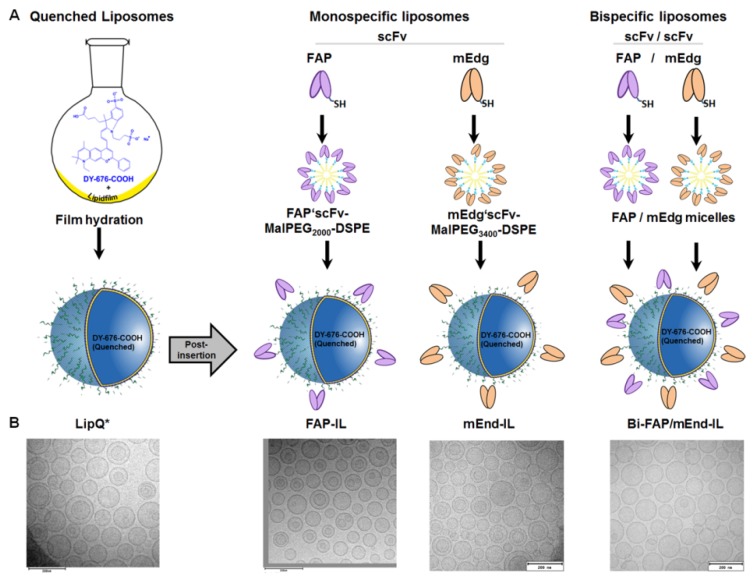
Scheme for the preparation of activatable bispecific fibroblast activation protein (FAP) and murine endoglin targeting liposomes. (**A**) The lipid film hydration was used to prepare liposomes (LipQ*) with high concentrations of quenched DY-676-COOH in the aqueous interior. Single chain antibody fragments (ScFv)-conjugated micelles specific for human FAP or murine endoglin were post-inserted in the quenched liposomes either individually to get the monospecific FAP-IL and mEnd-IL or simultaneously to get the bispecific Bi-FAP/mEnd-IL. Quenched Liposomes (LipQ) used as control were post-inserted with bare micelles. (**B**) Electron micrographs of the liposomes reveal a mix of unilamellar and a few multilamellar vesicles of approximately 100 nm diameter. Scale bar: 200 nm.

The NIRF dye DY-676-COOH, which exhibits self-quenching at high concentrations was used at a concentration of 4.3 mM–5 mM to hydrate a lipid film prior to extrusion through a 100 nm polycarbonate membrane. Liposomes formed in this way carried the dye in the aqueous interior in its fluorescence quenched state (Figure 1A, LipQ*). Thus, the fluorescence-quenched dye can be activated for example by liposomal degradation within cells, by disruption of liposome lipid bilayer with organic solvents or by crude handling conditions that lead to release of the dye and its dilution in the surrounding milieu. Starting with 5 mM concentrated DY-676-COOH solution, the encapsulation efficiency was in the range 4.6 to 6%. Nevertheless, the dye concentration within the liposomes was sufficiently quenched for the intended in vivo fluorescence imaging of tumors.

To achieve liposomal selectivity for cells expressing FAP, murine endoglin or both, MalPEG-micelles were pre-coupled to the respective specific scFv, then post-inserted into preformed, quenched liposomes (Figure 1A). Previous results revealed that the length of the PEG-chain influences the covalent conjugation, post insertion efficiency and stability of the resulting liposomes depending on the scFv [46]. This was the case with the murine endoglin specific scFv. For this reason, the MalPEG_2000_-DSPE micelles were used for the FAP’scFv, whereas the MalPEG_3400_-DSPE micelles were used for the murine endoglin scFv. The coupling efficiency for both FAP’scFv and mEdg’scFv to the micelles was higher than 90%, as could be deduced from gelelectrophoresis of the scFv protein before and after conjugation to the micelles (Supplementary Figure S1B). The amount of micelle lipids post-inserted into preformed liposomes varied for FAP and endoglin (0.1 mol% for FAP and 0.3 mol% for endoglin), and was chosen based on the stability and binding affinity of the resulting liposomes to target cells (Supplementary Figure S1C). To generate bispecific liposomes both micelles were post-inserted simultaneous (Figure 1A). Also, non-targeted quenched control liposomes (LipQ) were acquired by post-inserting cysteine-reduced MalPEG_3400_-DSPE to the LipQ* under similar conditions as for the targeted liposomes.

All the targeted liposomes showed minimal changes in the physical properties as compared to the non-targeted LipQ. These included slight increases in the vesicle sizes, DY-676-COOH concentration, and polydispersity indices (Table 1).

Interestingly, cryo-transmission electron microscopy revealed predominantly unilamellar vesicles and a few multilamellar ones (Figure 1B). The size data and polydispersity indices deduced by dynamic light scattering (Table 1) correlated well with observations from electron microscopy.

### 3.2. The Targeted and Control Liposomes Show Dye Activatability upon Damage

**Figure 2:**
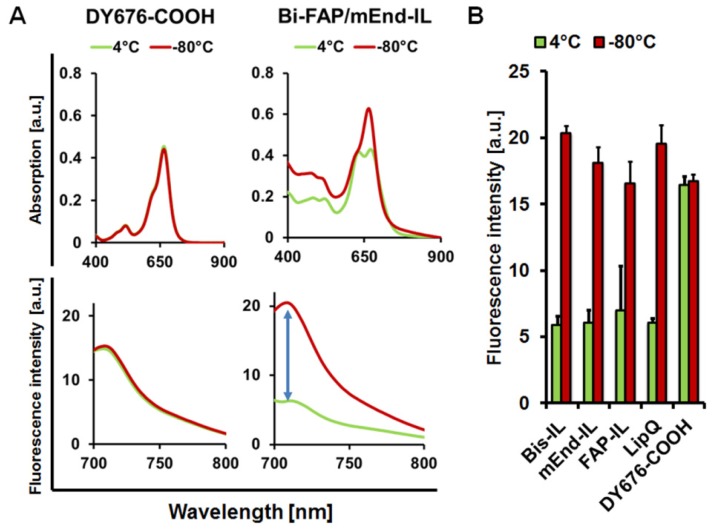
Spectroscopic validation of liposome quenching and activatability. (**A**) The free dye, DY-676-COOH at a concentration equivalent to the dye content in 100 nmol LipQ, gives a single absorption maximum and high fluorescence emission irrespective of storage at 4 °C or −80 °C. Contrarily, intact liposomes are fluorescence quenched and show a blue-shifted double absorption maximum and low fluorescence emission (4 °C, green lines). Upon freeze-damage at −80 °C the encapsulated DY-676-COOH is released and diluted in the surrounding solvent resulting in a single absorption maximum and almost 3-fold increase in fluorescence emission (red lines). (**B**) Semi-quantitative levels of fluorescence emission of intact (4 °C, green bars) and freeze-damaged liposomes (−80 °C, red bars). Each bar represents the mean of 3 independent measurements and standard deviation. Compared to the free dye all liposomes show significantly lower fluorescence when intact (*p* < 0.001) and an increase to almost the level of the free dye after activation.

Compared to the free dye which readily undergoes activation upon dilution in buffer, the intact liposomes showed fluorescence quenching of the encapsulated NIRF dye, DY-676-COOH upon dilution in buffer. This could be seen in a double absorption maximum with a blue shifted wavelength of the intact liposomes, and relatively low fluorescence emission, as demonstrated for the bispecific liposomal formulation (Figure 2A, *green lines*). Compared to the liposomes, free DY-676-COOH measured at a concentration equivalent to the DY-676-COOH content in 100 nmol (final lipids) of LipQ, showed a single absorption maximum and high fluorescence emission, which was not influenced by harsh freezing procedures. Opposed to the free DY-676-COOH, liposomal lipid membranes get damaged by harsh freezing processes, and consequently release the encapsulated cargoes into the surrounding solution. Consistently, diluting the liposomes in buffer after freeze-damaging resulted in fluorescence activation of the released dye, which was evident in a single absorption maximum that was shifted towards the red wavelengths, and also a 3-fold increase in fluorescence emission (Figure 2A, *red lines*). All the liposomes revealed comparable levels of fluorescence quenching and activation as opposed to the free dye. Interestingly, all the intact liposomal formulations showed significantly lower (°° *p* < 0.001) fluorescence intensities than the free DY-676-COOH (Figure 2B, *green bars*). Furthermore, a significant difference in fluorescence intensity (* *p* < 0.001) could be determined between the intact and freeze-damaged liposomes (Figure 2B). Opposed to this, the free DY-676-COOH stored at 4 °C or frozen at −80 °C did not show any difference in fluorescence. These observations substantiate the activation of the dye only upon release from the liposomes, and indicate their suitability in in vitro and in vivo applications whereby intact and fluorescence activated liposomes can be monitored.

### 3.3. Activatable Targeted Liposomes Are Selective for the Respective Target Cells In Vitro

**Figure 3:**
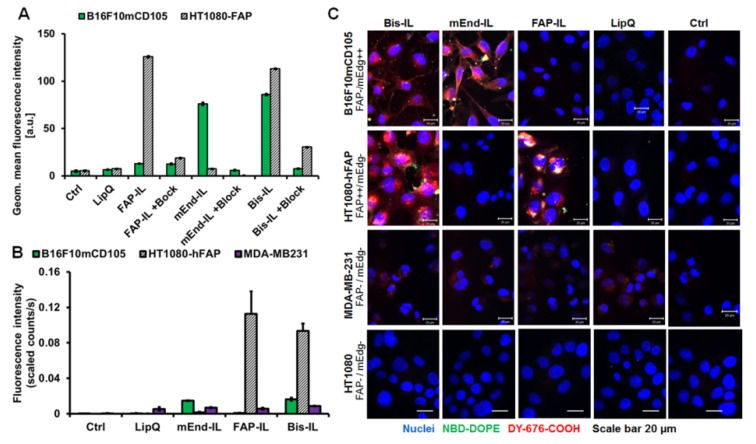
Selectivity of binding, uptake and activation of liposomes by cultured target cells. (**A**) Flow cytometric analysis of the liposomal green fluorescent phospholipid on the surface of FAP positive (HT1080-hFAP) and murine endoglin positive (B16F10mCD105) cells treated with the indicated liposomes alone or in the presence of free scFv as competitive blocker for 2 h at 4 °C. In the presence of the free FAP’scFv (+Block for the HT1080-hFAP) or free mEdg’scFv (+Block for the B16F10mCD105) binding is significantly (*p* < 0.001) reduced. Each bar represents the mean of 2 (with block) or 3 independent measurements and the standard deviation (S.D.). (**B**) Semi-quantitative levels of the fluorescence intensity of liposomal DY-676-COOH in cell pellets after incubation with 200 nmol (final lipids) of the respective liposomes for 8 h at 37 °C. Each bar depicts the mean of n = 3/SEM. Statistical significance (*t* test): *p* < 0.001 for FAP-IL and Bi-FAP/mEnd-IL versus mEnd-IL and LipQ in the HT1080-hFAP cells. (**C**) Confocal microscopic images of the indicated cell lines after treatment with 200 nmols (final lipids) of the indicated liposomes for 8 h at 37 °C.

To validate the ability of the liposomes to bind to the target receptors, they were added to human fibrosarcoma cells stably expressing high levels of human FAP (HT1080-hFAP) or murine melanoma cells stably expressing high levels of murine endoglin (B16F10mCD105). The cells were incubated at 4 °C for 2 h with the liposomes, either alone or in the presence of free scFv as competitive blocker, and then subsequently analyzed by flow cytometry. Under these energy-depleting conditions, the liposomes are not internalized and the NIRF DY-676-COOH is not activated. Hence, only the non-quenched green fluorescent phospholipid (NBD-DOPE) embedded in the liposomal lipid bilayer is detected. As seen in Figure 3A, only the Bi-FAP/mEnd-IL could bind to both FAP and murine endoglin on the HT1080-hFAP and the B16F10mCD105 cells. The FAP-IL and mEnd-IL could bind only to the HT1080-hFAP and the B16F10mCD105, respectively, indicating their specificity to the respective target receptors. Moreover, the binding of all targeted liposomes to the cells could be competitively blocked by the free scFv, whereas the non-targeted control LipQ revealed no binding to any of the cells, thus supporting the selectivity of the targeted liposomes for their target markers.

We further validated the internalization of the liposomes and subsequent activation of the encapsulated DY-676-COOH semi-quantitatively by NIRF imaging and also qualitatively by confocal microscopy. Besides the stable human FAP and murine endoglin expressing cells, the breast cancer cell line MDA-MB231 and the wild type of the fibrosarcoma cell line, HT1080, which lack expression of the FAP and murine endoglin proteins were also implemented. The cells incubated with the respective targeted probes for 8 h at standard culture conditions revealed high fluorescence intensities as opposed to the control LipQ (Figure 3B). Thus, the B16F10mCD105 cells selectively took up only the Bi-FAP/mEnd-IL and the mEnd-IL, whereas the HT1080-hFAP selectively took up only the Bi-FAP/mEnd-IL and the FAP-IL. In contrast, only negligible uptake was seen in the MDA-MB231 cells which lack target expression, and none of the cell lines took up the control LipQ (Figure 3B). The uptake and activation were reflected in the respective levels of semi-quantitative fluorescence intensities of cell pellets. The NIR fluorescence intensities in the HT1080-hFAP cell line were remarkably high, whereas those in the murine melanoma cell line B16F10mCD105 were comparably lower (Figure 3B, *blue bars*). This low level of fluorescence detection can be ascribed to interference of the fluorescence by melanin as previously shown in the melanoma cell line [47], especially when analyzed in a pelleted form as in the underlying setup. Consistently, analyses of the cells as monolayer cultures by confocal microscopy revealed strong fluorescence signals of liposomal DY-676-COOH in the murine melanoma (Figure 3C, *B16F10mCD105*). This was specific for the respective liposomal probes. Hence, the B16F10mCD105 revealed liposomal fluorescence only when they were treated with the Bi-FAP/mEnd-IL or the mEnd-IL, whereas the HT1080-hFAP cells revealed liposomal fluorescence signals only after incubation with the Bi-FAP/mEnd-IL or the FAP-IL (Figure 3C, *HT1080-hFAP*). In contrast, all liposomal formulations were not taken up by the wild type fibrosarcoma cell line, HT1080, whereas the human breast cancer cell line MDA-MB231 showed negligible levels of fluorescence of all the liposomes irrespective of targeting or not (Figure 3C, *MDA-MB231*). This suggests negligible interaction of liposomal lipids with the membrane of the breast cancer cells. Except for the negligible effect on MDA-MB231, the control LipQ were not taken up by any of the investigated cell lines. This supports the notion that targeted liposomes are taken up predominantly based on target-selective binding, and time dependent activation by the cells. This could be seen in time course monitoring of the uptake and activation process by confocal microscopy and NIRF imaging and semi-quantification of the fluorescence intensities (Supplementary data S2 and S3).

### 3.4. The Activatable Targeted Liposomes Show Distinct Biodistribution in Immune Deficient and Immune Competent Mice

**Figure 4:**
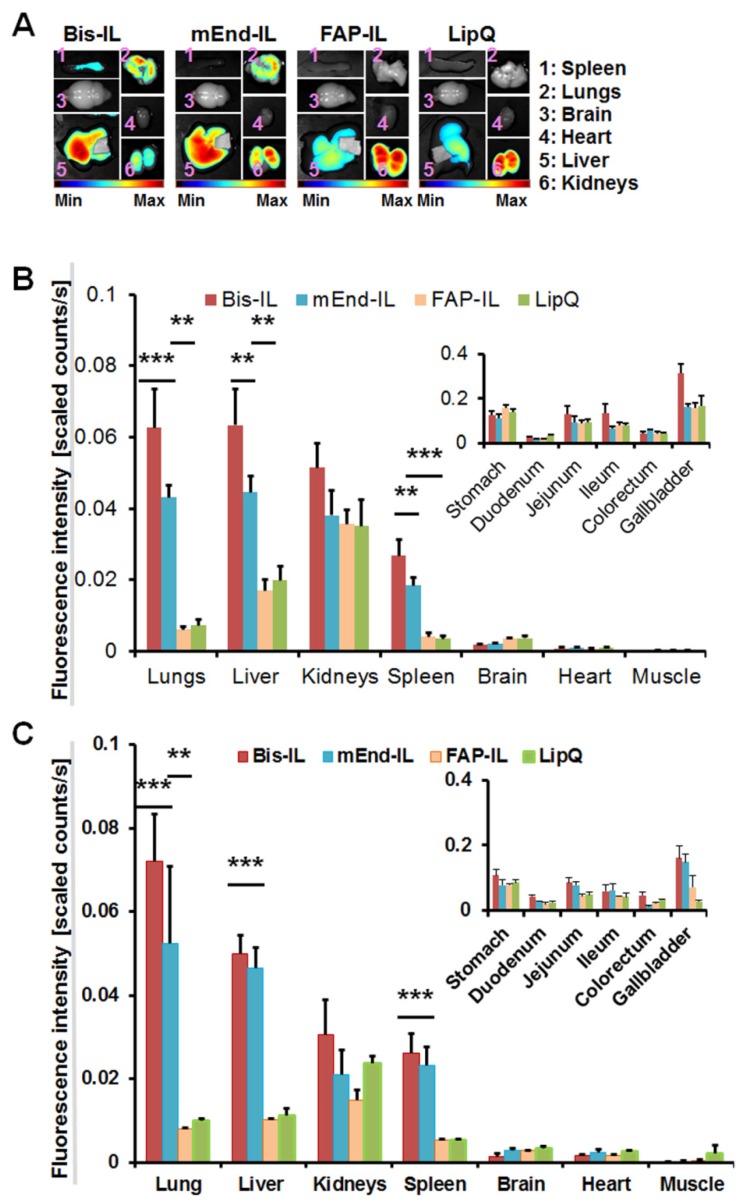
Biodistribution of liposomal formulations reveal target-specific alterations. (**A**) Representative near-infrared fluorescent (NIRF) images of organs from immune deficient athymic nude mice showing the DY-676-COOH signals 6 h p.i. of 20 µmol (final lipids)/kg body weight of the indicated liposomes. Semi-quantitative levels of the DY-676-COOH based fluorescence intensities of the respective organs excised from (**B**) immune deficient athymic nude mice, and (**C**) immune competent NMRI mice 6 h post injection. Inlays: gastrointestinal tract of the respective mice. The mEdg’scFv bearing liposomes (mEnd-IL and Bi-FAP/mEnd-IL, abbreviated Bis-IL), reveal a higher (*** *p* < 0.001 and ** *p* < 0.01) retention in the lungs, liver, and spleen than the FAP-IL and the control LipQ. Each bar depicts the mean of n = 3 mice and the SEM.

Previous data with the mEnd-IL revealed a high retention of the liposomal dye in the liver, kidneys and lungs of immune deficient mice by the first-pass effect. To know if this is the case in immune-competent mice, and also the influence of bispecific targeting of murine endoglin and FAP, the respective targeted and control liposomes were injected intravenously in female athymic nude mice (immune deficient) or male NMRI (immune competent) mice. The organs were excised and bio-optically imaged 6 h post injection as seen in the representative images of some organs isolated from the immune deficient nude mice (Figure 4A).

Except for a slight tendency to accumulate more in the kidneys of immune deficient mice, no differences in fluorescence intensities were detected in liver, kidneys lungs and spleen of the immune competent mice as compared to the immune deficient nude mice (Figure 4B,C). The mEnd-IL and the Bi-FAP/mEnd-IL showed very high accumulation in liver, lungs, kidneys and spleen, whereas the FAP-IL and control LipQ were predominantly seen in the liver, gall bladder and kidneys. At the investigated time point, FAP-IL and control LipQ revealed significantly lower fluorescence intensities (*p* < 0.001) than the Bi-FAP/mEnd-IL and the mEnd-IL. These differences in accumulation and distribution suggest characteristic interaction of the murine endoglin targeting liposomes with the vasculature of the mice organs, as seen in previous works [44]. Moreover, the Bi-FAP/mEnd-IL had higher fluorescence signals in most of the organs than the all the other liposomes (Figure 4, *red bars*), which suggests synergistic effects of the two antibody fragments on the biodistribution, retention and elimination of the liposomal components.

### 3.5. The Activatable Targeted Liposomes Show Distinct Accumulation in Tumor Models In Vivo

In the next step, we verified the ability of the bispecific liposomes to accumulate in xenografted human tumor models in mice and enable their fluorescence detection. The human fibrosarcoma HT1080-hFAP with high stable FAP expression, being known to produce large vasculature when xenografted in mice, and the breast carcinoma model MDA-MB231 with no FAP expression, but being known to develop high level of tiny vasculature in xenografted mice models were implemented. Mice bearing subcutaneously implanted tumors ranging in volume from 100 to 300 mm^3^ were injected intravenously with the bispecific, the monospecific and the control liposomes and then imaged by NIR fluorescence imaging over a period of 48 h. The tumor stroma including the tumor blood vessels and fibroblasts originate from the host (mouse) when human tumor cells are xenografted as reported herein. For this reason, the liposomes should be able to accumulate in the tumors based on distinct specificities to different components within the whole tumor. In this regard, the Bis-FAP/End-IL should accumulate in both tumor models based on binding to murine FAP expressing stromal cells such as myofibroblasts and pericytes and also to the murine endoglin expressing endothelial cells that make up the tumor vasculature. In the case of the HT1080-hFAP, whereby the tumor cells express human FAP, the liposomes bearing FAP’scFv (Bi-FAP/mEnd-IL and FAP-IL) should also accumulate in the tumor cells. Likewise, liposomes bearing the mEdg’scFv (Bi-FAP/mEnd-IL and mEnd-IL) should be taken up by the tumor vascular endothelial cells. Moreover, we could show that targeted liposomes can be taken up by phagocytosis though to a lesser degree than the non-targeted control LipQ. Hence all the liposomes should be taken up by tumor-associated macrophages. In accordance, the liposomes revealed varying accumulation patterns in the different tumor models.

#### 3.5.1. Liposome-Based Imaging of High FAP Expressing Tumor Models

**Figure 5:**
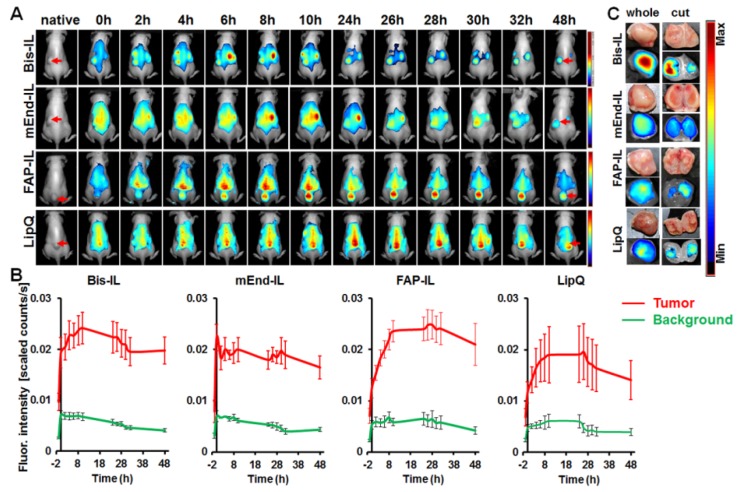
In vivo NIRF imaging of HT1080-hFAP tumors with activatable liposomes. (**A**) Representative NIRF images of mice imaged over 48 h post injection of 20 µmol (final lipids)/kg body weight of the indicated liposomes. Images per liposomal probe were compared with one another using the Maestro 3.0 software. Color-coded fluorescence: strong (red)-weak (blue). Red arrows depict the tumor. (**B**) Representative plots of the Semi-quantitative levels of the tumor fluorescence intensities after unmixing the autofluorescence. Each line depicts the mean of n = 7 (Bi-FAP/mEnd-IL), n = 3 (mEnd-IL), n = 5 (FAP-IL), n = 5 (LipQ) and the respective standard error, SEM. (**C**) Representative NIRF images of tumors excised from the mice 48 h post injection reveal retention of the liposomes.

In mice bearing the human fibrosarcoma, HT1080-hFAP tumors, the mEdg’scFv based liposomes (Bi-FAP/mEnd-IL and mEnd-IL) showed a rapid accumulation in the tumors within the initial minutes post application and a strong background fluorescence of the skin and organs of the mice (Figure 5A,B, *Bis-IL and mEnd-IL*). Thereby, a gradual increase in tumor fluorescence over time was peculiar in mice that received the bispecific liposome (Figure 5B, *Bis-IL*), whereas the tumor fluorescence in mice that received the mEnd-IL stagnated or decreased over time after injection (Figure 5A,B, *mEnd-IL*). The resulting tumor to background ratios (TBR) for the mEnd-IL group only reached 3 as from 24 h post injection and remained below 4 throughout the investigation period (Supplementary Figure S4A, *mEnd-IL*). Opposed to this, the TBR for the Bi-FAP/mEnd-IL mice group were above 3 as from 2 h post injection, and between 4 and 6 as from 24 h to 48 h (Supplementary Figure S4A, *Bis-IL*), which can be at least partially attributed to the FAP-binding component on the bispecific liposomes. Interestingly, the FAP-IL led to a gradual, but persistent increase in tumor fluorescence intensity over time (Figure 5A,B, *FAP-IL*), which resulted in TBRs in the range 4–6, as from 10 h post injection (Supplementary Figure S4A, *FAP-IL*). Likewise, the control LipQ caused a gradual and continuous increase in tumor fluorescence over time, but revealed a strong background fluorescence of the skin from the onset of injection (Figure 5A,B, *LipQ*). Semi-quantitative analyses of the fluorescence intensities revealed comparative levels for the control LipQ and the Bi-FAP/mEnd-IL (Figure 5A,B, *LipQ*). The resulting TBRs for the LipQ based imaging reached 3 as from 2 h post injection, but remained below 5 throughout the investigation period (Supplementary Figure S4A, *LipQ*), as opposed to the Bi-FAP/mEnd-IL which had TBRs above 5 at 48 h post injection. Except for the mEnd-IL which showed a significantly higher fluorescence intensity (*p* = 0.0192) than the FAP-IL shortly after injection (*t* = 0) the differences observed in the accumulation patterns between the liposomes were not statistically significant. There was only a slight tendency of the FAP-IL to cause higher fluorescence signals than the mEnd-IL and LipQ. Interestingly, based on the liposome formulation applied, the tumor fluorescence could be retained up to 48 h post injection when the background fluorescence had strongly reduced. Fluorescence images of the tumors excised at 48 h post injection revealed the most substantial retention of signals in the Bi-FAP/mEnd-IL group when compared with those excised from mice that received the other liposome formulations, using the Maestro 3.0 software tool (Figure 5C). This suggests a slower breakdown and release of the *Bi-FAP/mEnd-IL* based liposomal components.

#### 3.5.2. Liposome-Based Imaging of FAP/Endoglin Negative Tumor Models

**Figure 6:**
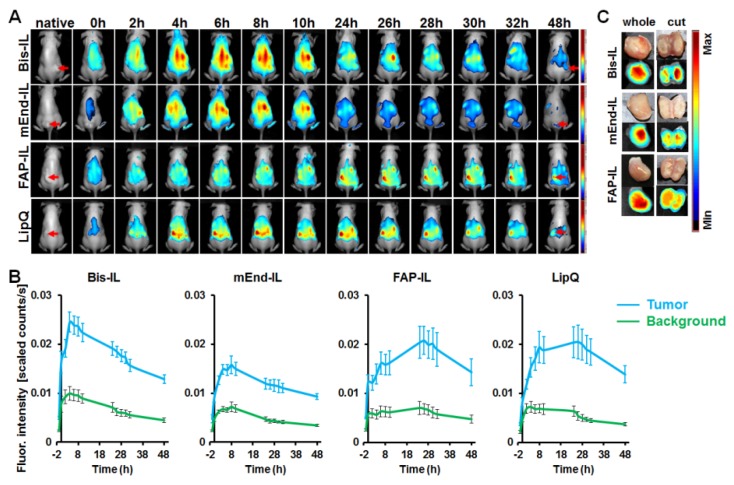
In vivo NIRF imaging of MDA-MB231 xenografts with activatable liposomes. (**A**) Representative NIRF images of mice imaged over 48 h post injection of 20 µmol (final lipids)/Kg body weight of the indicated liposomes. Images per liposomal probe were compared with one another using the Maestro 3.0 software. Color-coded fluorescence: strong (red)-weak (blue). Red arrows depict the tumor. (**B**) Representative plots of the semi-quantitative levels of the tumor fluorescence intensities after unmixing the background fluorescence. Each line depicts the mean of n = 7 (Bi-FAP/mEnd-IL), n = 7 (mEnd-IL), n = 5 (FAP-IL), n = 6 (LipQ) and the respective standard error, SEM. (**C**) Representative NIRF images of tumors excised from the mice 48 h post injection reveal retention of the liposomes.

Opposed to mice bearing the human fibrosarcoma (HT1080-hFAP) tumors, those bearing the human breast carcinoma (MDA-MB231) models showed further differences in the accumulation patterns of the liposomes. Hereby, the mEdg’scFv based liposomes (Bi-FAP/mEnd-IL and mEnd-IL) showed a rapid, but less persistent accumulation in the MDA-MB231 tumors shortly after application. A strong background fluorescence of the skin and organs of the mice was peculiar, whereas the tumor fluorescence reached a maximum already at 4 h and 8 h post injection for the Bi-FAP/mEnd-IL and mEnd-IL respectively (Figure 6A,B, *Bis-IL and mEnd-IL*), with the Bi-FAP/mEnd-IL group giving higher tumor signals than the mEnd-IL group (Figure 6B,C, *Bis-IL*). The tumor fluorescence signals dropped persistently thereafter. Contrarily, a gradual increase in tumor fluorescence over time was peculiar in mice that received the FAP-IL or control LipQ. Thereby, the tumor fluorescence in the FAP-IL group increased slowly over time and reached a maximum approximately 26 h after injection, before slightly decreasing in intensity (Figure 6A,B, *FAP-IL*). Opposed to the FAP-IL, the control LipQ signals in the MDA-MB231 already reached a maximum at 8 h post injection, then remained almost unchanged till 26 h, before dropping persistently (Figure 6A,B, *LipQ*). The TBR for the Bi-FAP/mEnd-IL and the mEnd-IL reached 3 as from 24 h post injection and did not increase further, whereas the TBR resulting from LipQ based imaging reached 3 as from 26 h post injection and increased only slightly thereafter (Supplementary Figure S4B). Likewise, the TBR resulting from FAP-IL based imaging reached 3 as from 24 h post injection and further increased slightly thereafter (Supplementary Figure S4B, *FAP-IL*).

A comparison of the fluorescence intensities in a semi-quantitative way revealed significant differences between the liposome-based fluorescence intensities at different time points after injection. Accordingly, the Bi-FAP/mEnd-IL revealed higher fluorescence intensities (*p* < 0.05) than the FAP-IL at 2 h–8 h, than the mEnd-IL at all the investigated time points (t = 0 h to 48 h) and also than LipQ at 0 h–6 h post injection. Interestingly, the FAP-IL and LipQ showed significantly higher tumor fluorescence levels than the mEnd-IL at 24–48 h post injection, suggesting a slower access of the targets (tumor-associated fibroblasts for the FAP-IL and macrophages for the LipQ) and hence a slower activation of the FAP-IL and LipQ in this tumor model. The respective differences in accumulation and distribution patterns of the liposomes in the respective tumor models further substantiate characteristic interaction of the liposomal formulations with distinct components of the mice tumors and organs. Furthermore, it exposes heterogeneity-related differences in the liposomal targeting of the tumor microenvironments of different tumor models.

### 3.6. Accumulation of Bispecific Liposomes in Tumors Is Based on Target Binding, Whereas Accumulation in Mice Liver and Kidneys Is Based on Both Target Binding and Elimination

**Figure 7:**
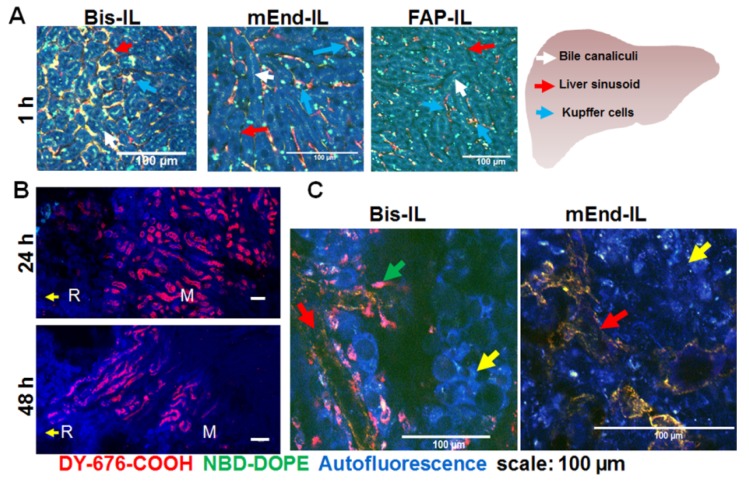
The bispecific liposomes localize in the liver within the sinusoids and Kupffer cells and in the tumors within the tumor vessels and myofibroblasts. (**A**) Microscopic images of fresh liver excised 60–70 min post injection. Bi-FAP/mEnd-IL (Bis-IL) signals locate predominantly in the sinusoids (*red arrow*), Kupffer cells (*blue arrow*) and bile canaliculi (*white arrow*), while the mEnd-IL fluorescence is seen in the sinusoids, and to a lower extent in Kupffer cells and bile canaliculi. FAP-IL signals are seen mainly in the Kupffer cells and bile canaliculi. (**B**) Representative microscopic image of the kidneys 24 h and 48 h post injection of Bi-FAP/mEnd-IL. “R” depicts the renal cortex and “M” the renal medulla. (**C**) Representative microscopic images of the MDA-MB231 xenograft showing Bi-FAP/mEnd-IL-based red fluorescence in the tumor vasculature (*red arrow*) and tumor myofibroblasts (*blue arrow*) and mEnd-IL based fluorescence only in the tumor vasculature. The tumor cells show only the tissue-specific blue autofluorescence (*yellow arrow*).

Since liposomal fluorescence was detectable in some organs of healthy and nude mice that did not bear tumors at 6 h post injection, and was also visible during whole body NIRF imaging of tumor bearing mice at longer time points post injection, it was vital to validate which cell subset the liposomal fluorescence was localized. Freshly excised liver tissues were imaged microscopically as reported previously [44]. The images revealed clearly distinguishable bile ducts in which both liposomal dyes were co-localized (Figure 7A). In addition, uptake of the liposomes into the Kupffer cells was seen. Furthermore, clear uptake of the mEdg’scFv based liposome formulations (Bi-FAP/mEnd-IL and mEnd-IL) into the liver sinusoid was evident. No uptake of the liposomes into the liver endothelium was found with FAP-IL (Figure 7A, *FAP-IL*).

When freshly excised kidneys were imaged microscopically at 24 h and 48 h post injection, the DY-676-COOH based fluorescence was detectable in the sections towards the cortex and the renal medulla at 24 h post injection (Figure 7B). Contrarily, a weaker fluorescence signal was detected from the medulla further towards the calyx at 48 h post injection, indicating a gradual elimination of the liposomal dye over time.

We further investigated the localization of the Bi-FAP/mEnd-IL based fluorescence signals in freshly isolated MDA-MB231 tumor xenografts. As mentioned earlier, the MDA-MB231 xenograft composed of a human breast cancer cell line which lack FAP and murine endoglin expression in vitro. Hence, in the in vivo situation, only the stromal fibroblasts which express murine FAP and the tumor endothelial cells which express the murine endoglin should take up and activate the liposomes. Consistently, confocal microscopy of freshly isolated MDA-MB231 tumor revealed Bi-FAP/mEnd-IL-based fluorescence exclusively in the tumor vasculature (Figure 7C, *red arrow*) and the tumor stromal fibroblasts, which could be recognized by their elongated morphology (Figure 7C, *green arrow*), but not the tumor cells (Figure 7C, *yellow arrow*). Opposed to this, mEnd-IL-based fluorescence signals are seen only in the tumor vasculature (Figure 7C, *red arrow*). This substantiates the selectivity of the Bi-FAP/mEnd-IL for its designated targets, namely murine endoglin on tumor endothelium and murine FAP on the tumor myofibroblasts and pericytes within this tumor model. As a reminder, the Bi-FAP/mEnd-IL carried the human FAP’scFv which reacts with both human- and murine FAP, due to a high amino acid sequence homology that exists between the human and murine FAP proteins.

### 3.7. The Activatable Bispecific Liposome Is Suitable for Intraoperative Detection

**Figure 8:**
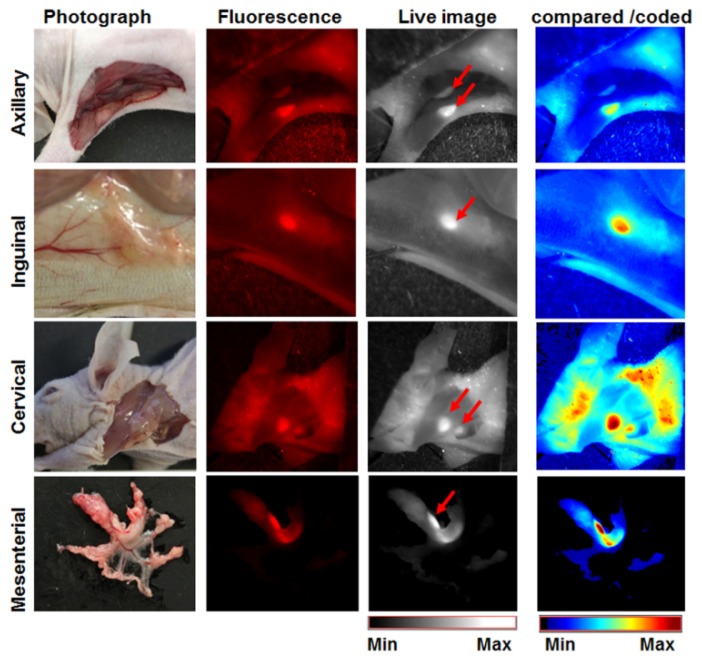
Bispecific liposomes enable demarcation of swollen lymph nodes in tumor-bearing mice. Mice bearing the HT1080-hFAP tumors were sacrificed and dissected 24 h post injection of 20 µmol (final lipids) Bi-FAP/mEnd-IL/kg body weight of the mice. The figure depicts representative photographs, fluorescence and live images showing the swollen axillary, inguinal, cervical and mesenterial lymph nodes, and the corresponding intensity scaled image acquired after comparison of the fluorescence intensities. Red arrows on the live images point at the clearly distinguishable lymph nodes.

The Bi-FAP/mEnd-IL accumulated in swollen lymph nodes and enhanced their detection during dissection. Accordingly, a strong fluorescence signal of swollen axillary, inguinal, cervical and mesenterial lymph nodes could be detected during dissection 24 h after injection of the Bi-FAP/mEnd-IL (Figure 8). As can be seen in the live images, the signals are clearly demarcated from the tissue autofluorescence. Thus, the observation suggests that the Bi-FAP/mEnd-IL could also be a useful tool for the image-guided delineation of tumors, metastatic lesions and suspicious lymph nodes during surgical interventions.

## 4. Discussion

### 4.1. Physical Properties of the Liposomal Probes

Liposomes are unique biocompatible nanocarriers that can be adapted very well for modifications tailored for defined applications in biomedical imaging and therapy, research on disease pathogenesis and therapy monitoring. We prepared bispecific liposomes encapsulated with a high concentration of a self-quenching NIRF dye, DY-676-COOH and endowed them with targeting antibody fragments directed towards the universal tumor markers FAP and endoglin. In previous work, we demonstrated the potentials of monospecific liposomes bearing the ligands specific to the individual targets. In the study presented here, we focused on elucidating the benefits of targeting these markers simultaneously. We used scFv as ligands, since they are price-efficient, easy to produce and guarantee target-selective binding while concurrently preventing possible immune responses that could be caused by whole antibodies [48]. The bispecific liposomes displayed increase in size as compared to bare quenched liposomes used for post-insertion, and exhibited average sizes of about 150 nm as opposed to non-targeted and monospecific liposomes (LipQ, FAP-IL and mEnd-IL) which exhibited diameters in the range 130–140 nm. The polydispersity indices were below 0.3, indicating a relative uniformity of the vesicles, whereas the zeta potentials revealed a negative charge of the vesicles. The Bi-FAP/mEnd-IL stably retained the encapsulated DY-676-COOH when stored at 4 °C, and was fluorescence quenched. Moreover, its red fluorescence was exclusively activated upon triggering release and subsequent activation of the dye, for example in buffer or within target cells. Spectroscopic analyses revealed characteristic blue-shifts in the absorption maxima and low fluorescence emissions which is indicative of fluorescence quenching [49,50], whereas freeze-damaging of the liposomes by storing at −80 °C resulted in a red shifted single absorption maximum and almost 3 fold increase in fluorescence emission which signifies activation. The intrinsic quenching of the DY-676-COOH results from π-stacking interaction and fluorescence resonance energy transfer (FRET) which occurs at concentrations where dye donor and acceptor species lie at molecule distances below 100Å [51]. This phenomenon is seen in many fluorophores [49], and is reversible during dilution of fluorophores in buffers, organic solvents [33] or protic environments that increase the intramolecular distance between the fluorophore species. Thus, fluorescence quenching and activation of the Bi-FAP/mEnd-IL could be easily exploited for in this work for imaging purposes as reported earlier for monospecific liposomes [12,35,50].

### 4.2. Selectivity of Bispecific Liposomes to the Targets on Cultured Cells

The Bi-FAP/mEnd-IL was a highly selective for cultured cells that expressed either FAP or murine endoglin, but not for target negative cells. This was evident in its exclusive uptake by the HT1080-hFAP and B16F10mCD105 cells. In contrast, the monospecific FAP-IL and mEnd-IL was taken up only by the HT1080-hFAP and the B16F10mCD105 cells, respectively, whereas LipQ was not taken up by any of the cancer cell lines. Target binding was evident in the high levels of green fluorescence signals of cells acquired by flow cytometry after incubation with the liposomes at energy depleting conditions, and also in the reduced fluorescence intensity seen after competitive blocking with the respective free scFv. Furthermore, liposomal activation was substantiated after incubation with cells under standard culture conditions. Except for the murine melanoma B16F10mCD105, which produces high levels of melanin that was shown to interfere with NIR fluorescence imaging of the cell pellets [47,52,53,54] cell-related fluorescence activation of the liposomes could be validated semi-quantitatively and correlated well with the qualitative visualization by confocal microscopy.

Thereby, key differences were observed between Bi-FAP/mEnd-IL and FAP-IL in the kinetics of FAP-based binding, uptake and activation, but not for endoglin-based binding and activation. Thus, a slower uptake and activation of the Bi-FAP/mEnd-IL was seen in time course experiments on the HT1080-hFAP (Supplementary data S2), which suggests a partial shielding of the FAP-binding moiety on the liposomes by the mEdg’scFv which is coupled to longer PEG_3400_ chains on the Bi-FAP/mEnd-IL surface. A comparable influence of liposomal PEG-pairs with different molecular weights on target binding of the resulting liposomes was observed by other researchers [55]. However, the shielding did not abolish FAP detection by the Bi-FAP/mEnd-IL, but only reduced the speed, as evident in the persistent increase in Bi-FAP/mEnd-IL based NIR fluorescence at 24 h post incubation with cells. In comparison, FAP-IL based NIR fluorescence reached a maximum already after 12 h incubation with cells (Supplementary Figure S2C). Opposed to the FAP-based targeting, the Bi-FAP/mEnd-IL retained binding to murine endoglin expressing cells and subsequent fluorescence activation at a comparable level to the monospecific mEnd-IL (Supplementary data S3). This further supports the fact that a partial shielding of FAP-binding moiety is the cause of the retarded binding of Bi-FAP/mEnd-IL to FAP-expressing target cells. Interestingly, the targeted and control liposomes were not taken up by the target negative breast carcinoma MDA-MB231 or the wild type fibrosarcoma HT1080 cell lines, and further indicate that the targeted liposomes are highly selective for their targets. In previous works, we showed evidence of a predominant phagocytic uptake of the non-targeted, quenched LipQ [33] and also a reduced, but possible phagocytic uptake of ligand targeted liposomal formulations by cultured macrophages [35,45,46]. The observation suggests that all the liposomal formulations should be taken up to some extent by tumor associated macrophages in the in vivo situation.

### 4.3. Biodistribution, Selectivity and Potential Applications of Bispecific Liposomes In Vivo

Fluorescence quenching and activatability of the bispecific and control liposomes could be exploited to reliably assess the potentials and comparative limitations in in vivo studies. This was merit to the fact that intact quenched liposomes that access the tumors by EPR-effect alone are not detected by NIR-fluorescence imaging, but after uptake and degradation by cells, get activated and enhance NIR fluorescence with high sensitivity, thereby enabling detection of the structures into which they accumulate. Opposed to the target selectivity of the liposomes in cell culture studies, the in vivo situation of xenografted human cancer cells in mice present more candidate target structures, whereby the tumor stroma originates from the mice. In this respect, the level of many components of the tumor microenvironment, including the tumor associated macrophages (TAMs), tumor vascular density and tumor associated fibroblasts (TAFs) vary between tumor types, and hence impact the targeting of the tumors with drugs or imaging probes. Thus, we expected the Bi-FAP/mEnd-IL to simultaneously target the tumor cells (based on human FAP), TAFs (based on murine FAP of host mouse) [5], tumor vasculature (based on murine endoglin) [32] and TAMs (based on phagocytic uptake) [33]. Many reports suggest that the diameter of nanomaterials for a successful leakage from tumor vessels and internalization into tumor cells and the tumor microenvironment should be in the range of 10 nm to 100 nm [56]. However, the Bi-FAP/mEnd-IL with a size of 150 nm diameter, and the monospecific and control liposomes with diameters between 130–140 nm could accumulate in the tumors and enhance their imaging, which indicates an additional benefit of molecular targeting over dependence on leakiness by passive delivery approaches.

Consistently, the liposomes accumulated in tumor models originating from human FAP-expressing fibrosarcoma cells and the FAP- and murine endoglin negative breast carcinoma cells at varying levels as could be seen in the differences in fluorescence intensities of the tumors over time post injection. Compared to the monospecific liposomes (FAP-IL and mEnd-IL) the accumulation pattern of the Bi-FAP/mEnd-IL clearly exposed a synergy of the targeting moieties. This was strongly related to the tumor model in question, with the murine endoglin binding moiety showing a partial dominant impact than the FAP binding moiety. As seen in cell culture studies, this can partly be due to the size of the PEG3400 to which the mEdg’scFv is conjugated. Thus, the overall accumulation of the Bi-FAP/mEnd-IL in the FAP-expressing tumor model increased persistently over time and was still very high 48 h post injection. This is in contrast to the double negative MDA-MB231 breast cancer model which revealed a rapid accumulation of the Bi-FAP/mEnd-IL and a persistent decease of the tumor fluorescence as from 4 h post injection onwards (compare Figure 5B and Figure 6B, *Bis-IL*). Interestingly, the mEnd-IL revealed a higher accumulation in the fibrosarcoma model than in the breast carcinoma model and exposed a relatively similar time-based accumulation pattern like the Bi-FAP/mEnd-IL, but with overall lower fluorescence intensities over time. In contrast, FAP-IL and LipQ accumulated slowly and persistently in both tumor models, whereby higher fluorescence signals were seen in the fibrosarcoma model. Thus, the accumulation patterns also reflected the depth of the target structures away from the vasculature. Therefore, it is not surprising that only the Bi-FAP/mEnd-IL and the mEnd-IL accumulated rapidly and are eliminated persistently in the MDA-MB231 breast cancer model where larger amounts of small sized blood vessels are present, as compared to the fibrosarcoma model with lower vessel burden and large vessel lumen as reported previously [46]. The presence of the targets directly on the tumor vasculature guarantees a rapid detection and binding as seen with the Bi-FAP/mEnd-IL and the mEnd-IL in this tumor model. Contrarily, the location of the tumor cells, TAFs and TAMs further away from the tumor vasculature accounts for a much slower and persistent accumulation as seen for the Bi-FAP/mEnd-IL, FAP-IL and LipQ, but not the mEnd-IL in the FAP-expressing fibrosarcoma model (HT1080-hFAP) and also for the FAP-IL and LipQ in the breast carcinoma (MDA-MB231) model. Although Pauuwe and colleagues reported the expression of endoglin by TAFs in colon carcinoma [57], we could not see this in our tumor models from mEnd-IL injected mice. This suggests that endoglin expression by TAFs may be negligible in our models, or is tumor type specific, similar to the expression of endoglin on a selected number of neoplastic cells.

Interestingly, a high accumulation of the Bi-FAP/mEnd-IL and mEnd-IL was seen in the liver, lungs and kidneys of immune deficient and immune competent mice, whereby no significant difference could be deduced between the mice strains. This accumulation was based on liposomal clearance as reported for FAP-IL and LipQ previously [5,33], and on binding of murine endoglin that is expressed in the vasculature of mice lungs, liver and kidneys [44,46], as evident for example in microscopic images of freshly excised liver tissues from mice that received the targeted liposomes (Figure 7A). Elimination of liposomal components accounts for a predominant localization of liposomal fluorescence in the bile canaliculi/liver Kupffer cells and their decrease over time in kidney tubules as observed with the Bi-FAP/mEnd-IL at 24 h and 48 h post injection (Figure 7B). Although the binding of endoglin in the mice organs especially during the first-pass after injection greatly reduced the level of Bi-FAP/mEnd-IL and mEnd-IL that reached the tumors, the observation strongly substantiates the specificity of the liposomes for specific target cells. In effect the Bi-FAP/mEnd-IL based fluorescence was localized only in the tumor vasculature and myofibroblasts of the breast carcinoma model (Figure 7C).

Although, the binding of the Bi-FAP/mEnd-IL to the vascular endothelia of mice lungs, liver and kidneys was related to the overexpression of murine endoglin and hence contributed to an overall comparable tumor fluorescence levels to that seen with FAP-IL, a more suitable and reliable tumor imaging based on the simultaneous targeting of human endoglin and human FAP should be expected. Liposomes are readily modifiable [58], hence replacing the mEdg’scFv with human Edg’scFv is easy to realize. Furthermore, the pattern of endoglin expression in healthy human tissues greatly varies from that seen in mice in the underlying and previous studies [44,46]. It is known that the microvasculatures of some healthy human tissues have basal expression of endoglin, which is never-the-less negligible in the liver, kidneys and lungs [59] that contribute immensely in the first-pass entrapment of intravenously injected substances. The overexpression of human endoglin is restricted to healing wounds, developing embryos, inflamed tissues, some neoplastic cells and the tumor vascular cells of many solid tumors [32]. Thus, a Bi-FAP/mEnd-IL formulation tailored for the targeting of human endoglin and FAP will be suitable for the targeted delivery of drugs and contrast agents into human neoplastic cells, and the tumor microenvironment components including the TAFs, tumor vasculature and also TAMs. Preliminary in vitro studies demonstrated the feasibility of loading bispecific FAP and endoglin targeting liposomes with doxorubicin [60], indicating that a potential modification of the bispecific liposomes for targeted delivery of a variety of different drugs should be feasible.

Furthermore, the Bi-FAP/mEnd-IL accumulated into swollen suspicious lymph nodes that could be clearly demarcated during intra-operative simulations by live NIRF imaging (Figure 8). Hence, their use in image-guided detection of tumors, invaded tumor margins and metastases during surgical interventions would also be feasible.

## 5. Conclusions

Similar to the monospecific liposomes, the Bi-FAP/mEnd-IL represents a potent tool for the demarcation of invaded tumor margins and suspicious lymph nodes during intra-operative procedures. Compared to non-targeted or monospecific liposomes, the advantage of bispecific FAP and endoglin targeting liposomes does not lie in increasing the overall level of cargo in the tumors per unit time per se, but in the precise delivery of the cargos into the distinct tumor stromal fibroblasts and tumor vascular endothelial cells simultaneously. Thus, the results suggest that the modification with human endoglin targeting scFv and the replacement of the encapsulated dye with therapeutic cargos such as chemotherapeutic drugs, nucleotides or immune modulating therapeutics is a promising approach for the future modulation of the tumor stroma of cancer patients by simultaneous targeting of FAP and endoglin.

## Figures and Tables

**Table 1:** Properties of dye-loaded activatable liposomes.

Parameter	Z-Average [nm]	PDI	Zeta Potential [mV]	DY-676 (µg/mL)
LipQ *	128.1 ± 2	0.072 ± 0.02	−10.9 ± 0.7	161
LipQ	133.0 ± 1	0.081 ± 0.10	−17.0 ± 1.1	152
mEnd-IL	137.0 ± 2	0.180 ± 0.01	−13.0 ± 0.4	158
FAP-IL	139.1 ± 2.5	0.079 ± 0.02	−15.4 ± 4.9	148
Bi-FAP/mEnd-IL	154.5 ± 5.3	0.107 ± 0.02	−20.5 ± 0.64	178

* Preformed quenched liposomes which were fused to cysteine-reduced micelles to get LipQ (control) or to ligand conjugated micelles to get mEnd-IL, FAP-IL and the Bi-FAP/mEnd-IL.

## Supplementary Materials

The following are available online at https://www.mdpi.com/1999-4923/12/4/370/s1, Figure S1: (**A**) Representative control analysis of the scFv melting temperature, (**B**) Polyacrylamide gelectrophoresis analysis of the scFv proteins under non-denaturing conditions, before “1” and after conjugation to micelles “2”. The letters *a* and *a** depict the free and micelle-conjugated scFv bands, respectively. (**C**) Concentration-dependent binding of targeted liposomes to FAP (upper graph) and murine endoglin (lower graph) expressing target cells, after the insertion of micelles at varying concentrations. Using 0.1 mol% FAP’scFv micelles resulted in the highest binding to the target HT1080-FAP cells (green line), whereas post insertion of 0.3 mol% mEdg’scFv micelles showed suitable binding of liposomes to target B16F10-mCD105 cells (red line), Figure S2: (**A**) Confocal microscopic images of HT1080-hFAP cells showing the characteristic fluorescence of liposomal green-fluorescent phospholipid NBD-DOPE and red fluorescence of the encapsulated DY-676-COOH. (**B**). Representative DY 676-COOH based NIRF images of cell pellets overlaid with the white light images after incubation of the cells with liposomes for the indicated durations. (**C**) Corresponding semi-quantitative levels of the DY-676-COOH based fluorescence intensities of the cell pellets. Each line depicts the mean of the fluorescence intensities of 2 independent experiments at the given time points and their standard deviations. No difference (*p* > 0.05) could be determined between the mEnd-IL based fluorescence levels at the given time points, Figure S3: (**A**) Confocal microscopic images of B16F10mCD105 cells showing the characteristic fluorescence of the liposomal green-fluorescent phospholipid NBD-DOPE, and the red fluorescence of the encapsulated DY-676-COOH. (**B**). Representative DY-676-COOH based NIRF images of cell pellets overlaid with the white light images after incubation of the cells with liposomes for the indicated durations. (**C**) Corresponding semi-quantitative levels of the DY-676-COOH based fluorescence intensities of the cell pellets. Each line depicts the mean of the fluorescence intensities of 2 independent experiments at the given time points and their standard deviations. No difference (*p* > 0.05) could be determined between the mEnd-IL based fluorescence levels at the given time points, Figure S4: Tumor to background ratios of the fluorescence intensities detected in the high FAP-expressing HT1080-hFAP and the target negative MDA-MB231 based xenografts. For the HT1080-hFAP, each bar represents the mean of n = 7 (Bis-IL), n = 3 (mEnd-IL), n = 5 (FAP-IL), n = 5 (LipQ), and the respective SEM. For the MDA-MB231, each bar represents the mean of n = 7 (Bis-IL), n = 7 (mEnd-IL), n = 5 (FAP-IL), n = 6 (LipQ), and the respective SEM.

## References

[1] Fahr A., Liu X. (2007). Drug delivery strategies for poorly water-soluble drugs. Expert Opin. Drug Deliv..

[2] Aryasomayajula B., Salzano G., Torchilin V.P. (2017). Multifunctional Liposomes. Methods Mol. Biol..

[3] Fahr A., van Hoogevest P., May S., Bergstrand N., ML S.L. (2005). Transfer of lipophilic drugs between liposomal membranes and biological interfaces: Consequences for drug delivery. Eur. J. Pharm. Sci. Off. J. Eur. Fed. Pharm. Sci..

[4] Gabizon A., Shmeeda H., Grenader T. (2012). Pharmacological basis of pegylated liposomal doxorubicin: Impact on cancer therapy. Eur. J. Pharm. Sci. Off. J. Eur. Fed. Pharm. Sci..

[5] Rüger R., Tansi F.L., Rabenhold M., Steiniger F., Kontermann R.E., Fahr A., Hilger I. (2014). In vivo near-infrared fluorescence imaging of FAP-expressing tumors with activatable FAP-targeted, single-chain Fv-immunoliposomes. J. Control. Release Off. J. Control. Release Soc..

[6] Khorana A.A., Ryan C.K., Cox C., Eberly S., Sahasrabudhe D.M. (2003). Vascular endothelial growth factor, CD68, and epidermal growth factor receptor expression and survival in patients with Stage II and Stage III colon carcinoma: A role for the host response in prognosis. Cancer.

[7] Funada Y., Noguchi T., Kikuchi R., Takeno S., Uchida Y., Gabbert H.E. (2003). Prognostic significance of CD8+ T cell and macrophage peritumoral infiltration in colorectal cancer. Oncol. Rep..

[8] Zhou Q., Peng R.Q., Wu X.J., Xia Q., Hou J.H., Ding Y., Zhou Q.M., Zhang X., Pang Z.Z., Wan D.S. (2010). The density of macrophages in the invasive front is inversely correlated to liver metastasis in colon cancer. J. Transl. Med..

[9] Ferrari M. (2005). Cancer nanotechnology: Opportunities and challenges. Nat. Rev. Cancer.

[10] Gottesman M.M., Fojo T., Bates S.E. (2002). Multidrug resistance in cancer: Role of ATP-dependent transporters. Nat. Rev. Cancer.

[11] Adams G.P., Schier R., McCall A.M., Simmons H.H., Horak E.M., Alpaugh R.K., Marks J.D., Weiner L.M. (2001). High affinity restricts the localization and tumor penetration of single-chain fv antibody molecules. Cancer Res..

[12] Li J., Chen K., Liu H., Cheng K., Yang M., Zhang J., Cheng J.D., Zhang Y., Cheng Z. (2012). Activatable near-infrared fluorescent probe for in vivo imaging of fibroblast activation protein-alpha. Bioconjugate Chem..

[13] Park J.W., Hong K., Kirpotin D.B., Colbern G., Shalaby R., Baselga J., Shao Y., Nielsen U.B., Marks J.D., Moore D. (2002). Anti-HER2 immunoliposomes: Enhanced efficacy attributable to targeted delivery. Clin. Cancer Res. Off. J. Am. Assoc. Cancer Res..

[14] Laginha K., Mumbengegwi D., Allen T. (2005). Liposomes targeted via two different antibodies: Assay, B-cell binding and cytotoxicity. Biochim. Biophys. Acta.

[15] Kalluri R., Zeisberg M. (2006). Fibroblasts in cancer. Nat. Rev. Cancer.

[16] Bauer S., Jendro M.C., Wadle A., Kleber S., Stenner F., Dinser R., Reich A., Faccin E., Godde S., Dinges H. (2006). Fibroblast activation protein is expressed by rheumatoid myofibroblast-like synoviocytes. Arthritis Res. Ther..

[17] Brennen W.N., Isaacs J.T., Denmeade S.R. (2012). Rationale behind targeting fibroblast activation protein-expressing carcinoma-associated fibroblasts as a novel chemotherapeutic strategy. Mol. Cancer Ther..

[18] Kelly T. (2005). Fibroblast activation protein-alpha and dipeptidyl peptidase IV (CD26): Cell-surface proteases that activate cell signaling and are potential targets for cancer therapy. Drug Resist. Updates Rev. Comment. Antimicrob. Anticancer Chemother..

[19] Zhi K., Shen X., Zhang H., Bi J. (2010). Cancer-associated fibroblasts are positively correlated with metastatic potential of human gastric cancers. J. Exp. Clin. Cancer Res. CR.

[20] Hofheinz R.D., al-Batran S.E., Hartmann F., Hartung G., Jager D., Renner C., Tanswell P., Kunz U., Amelsberg A., Kuthan H. (2003). Stromal antigen targeting by a humanised monoclonal antibody: An early phase II trial of sibrotuzumab in patients with metastatic colorectal cancer. Onkologie.

[21] Jiang G.M., Xu W., Du J., Zhang K.S., Zhang Q.G., Wang X.W., Liu Z.G., Liu S.Q., Xie W.Y., Liu H.F. (2016). The application of the fibroblast activation protein alpha-targeted immunotherapy strategy. Oncotarget.

[22] Huang Y., Simms A.E., Mazur A., Wang S., Leon N.R., Jones B., Aziz N., Kelly T. (2011). Fibroblast activation protein-alpha promotes tumor growth and invasion of breast cancer cells through non-enzymatic functions. Clin. Exp. Metastasis.

[23] Lee H.O., Mullins S.R., Franco-Barraza J., Valianou M., Cukierman E., Cheng J.D. (2011). FAP-overexpressing fibroblasts produce an extracellular matrix that enhances invasive velocity and directionality of pancreatic cancer cells. BMC Cancer.

[24] Klein-Goldberg A., Maman S., Witz I.P. (2013). The role played by the microenvironment in site-specific metastasis. Cancer Lett..

[25] Santos A.M., Jung J., Aziz N., Kissil J.L., Pure E. (2009). Targeting fibroblast activation protein inhibits tumor stromagenesis and growth in mice. J. Clin. Investig..

[26] Ge A.Z., Butcher E.C. (1994). Cloning and expression of a cDNA encoding mouse endoglin, an endothelial cell TGF-beta ligand. Gene.

[27] Bernabeu C., Lopez-Novoa J.M., Quintanilla M. (2009). The emerging role of TGF-beta superfamily coreceptors in cancer. Biochim. Biophys. Acta.

[28] Aird W.C. (2009). Molecular heterogeneity of tumor endothelium. Cell Tissue Res..

[29] Gregory A.L., Xu G., Sotov V., Letarte M. (2014). Review: The enigmatic role of endoglin in the placenta. Placenta.

[30] Fonsatti E., Altomonte M., Arslan P., Maio M. (2003). Endoglin (CD105): A target for anti-angiogenetic cancer therapy. Curr. Drug Targets.

[31] Fonsatti E., Jekunen A.P., Kairemo K.J., Coral S., Snellman M., Nicotra M.R., Natali P.G., Altomonte M., Maio M. (2000). Endoglin is a suitable target for efficient imaging of solid tumors: In vivo evidence in a canine mammary carcinoma model. Clin. Cancer Res. Off. J. Am. Assoc. Cancer Res..

[32] Dallas N.A., Samuel S., Xia L., Fan F., Gray M.J., Lim S.J., Ellis L.M. (2008). Endoglin (CD105): A marker of tumor vasculature and potential target for therapy. Clin. Cancer Res. Off. J. Am. Assoc. Cancer Res..

[33] Tansi F.L., Rüger R., Rabenhold M., Steiniger F., Fahr A., Kaiser W.A., Hilger I. (2013). Liposomal Encapsulation of a Near-Infrared Fluorophore Enhances Fluorescence Quenching and Reliable Whole Body Optical Imaging Upon Activation In Vivo. Small.

[34] Tansi F.L., Rüger R., Rabenhold M., Steiniger F., Fahr A., Hilger I. (2015). Fluorescence-quenching of a liposomal-encapsulated near-infrared fluorophore as a tool for in vivo optical imaging. J. Vis. Exp. Jove.

[35] Tansi F.L., Rüger R., Böhm C., Kontermann R.E., Teichgraeber U.K., Fahr A., Hilger I. (2016). Potential of activatable FAP-targeting immunoliposomes in intraoperative imaging of spontaneous metastases. Biomaterials.

[36] Brocks B., Garin-Chesa P., Behrle E., Park J.E., Rettig W.J., Pfizenmaier K., Moosmayer D. (2001). Species-crossreactive scFv against the tumor stroma marker “fibroblast activation protein” selected by phage display from an immunized FAP-/- knock-out mouse. Mol. Med..

[37] Matsuno F., Haruta Y., Kondo M., Tsai H., Barcos M., Seon B.K. (1999). Induction of lasting complete regression of preformed distinct solid tumors by targeting the tumor vasculature using two new anti-endoglin monoclonal antibodies. Clin. Cancer Res. Off. J. Am. Assoc. Cancer Res..

[38] Rüger R., Müller D., Fahr A., Kontermann R.E. (2005). Generation of immunoliposomes using recombinant single-chain Fv fragments bound to Ni-NTA-liposomes. J. Drug Target..

[39] Müller D., Trunk G., Sichelstiel A., Zettlitz K.A., Quintanilla M., Kontermann R.E. (2008). Murine endoglin-specific single-chain Fv fragments for the analysis of vascular targeting strategies in mice. J. Immunol. Methods.

[40] Müller D., Karle A., Meissburger B., Höfig I., Stork R., Kontermann R.E. (2007). Improved pharmacokinetics of recombinant bispecific antibody molecules by fusion to human serum albumin. J. Biol. Chem..

[41] Iden D.L., Allen T.M. (2001). In vitro and in vivo comparison of immunoliposomes made by conventional coupling techniques with those made by a new post-insertion approach. Biochim. Biophys. Acta.

[42] Allen T.M., Sapra P., Moase E. (2002). Use of the post-insertion method for the formation of ligand-coupled liposomes. Cell. Mol. Biol. Lett..

[43] Feldman J.P., Goldwasser R., Mark S., Schwartz J., Orion I. (2009). A Mathematical Model for Tumor Volume Evaluation using Two-Dimensions. J. Appl. Quant. Methods.

[44] Tansi F.L., Rüger R., Kollmeier A.M., Böhm C., Kontermann R.E., Teichgraeber U.K., Fahr A., Hilger I. (2017). A fast and effective determination of the biodistribution and subcellular localization of fluorescent immunoliposomes in freshly excised animal organs. BMC Biotechnol..

[45] Tansi F.L., Rüger R., Böhm C., Steiniger F., Kontermann R.E., Teichgraeber U.K., Fahr A., Hilger I. (2017). Activatable bispecific liposomes bearing fibroblast activation protein directed single chain fragment/Trastuzumab deliver encapsulated cargo into the nuclei of tumor cells and the tumor microenvironment simultaneously. Acta Biomater..

[46] Tansi F.L., Ruger R., Kollmeier A.M., Rabenhold M., Steiniger F., Kontermann R.E., Teichgraeber U.K., Fahr A., Hilger I. (2018). Endoglin based in vivo near-infrared fluorescence imaging of tumor models in mice using activatable liposomes. Biochim. Biophys. Acta Gen. Subj..

[47] Tansi F.L., Ruger R., Kollmeier A.M., Rabenhold M., Steiniger F., Kontermann R.E., Teichgraeber U.K., Fahr A., Hilger I. (2018). Dataset on the role of endoglin expression on melanin production in murine melanoma and on the influence of melanin on optical imaging. Data Brief.

[48] Thorpe S.J., Turner C., Heath A., Feavers I., Vatn I., Natvig J.B., Thompson K.M. (2003). Clonal analysis of a human antimouse antibody (HAMA) response. Scand. J. Immunol..

[49] Ogawa M., Kosaka N., Choyke P.L., Kobayashi H. (2009). H-type dimer formation of fluorophores: A mechanism for activatable, in vivo optical molecular imaging. ACS Chem. Biol..

[50] Johansson M.K., Cook R.M. (2003). Intramolecular dimers: A new design strategy for fluorescence-quenched probes. Chemistry.

[51] Wu P., Brand L. (1994). Resonance energy transfer: Methods and applications. Anal. Biochem..

[52] Weissleder R., Ntziachristos V. (2003). Shedding light onto live molecular targets. Nat. Med..

[53] Zonios G., Dimou A. (2008). Melanin optical properties provide evidence for chemical and structural disorder in vivo. Opt. Express.

[54] Ito A.S., Azzellini G.C., Silva S.C., Serra O., Szabo A.G. (1992). Optical absorption and fluorescence spectroscopy studies of ground state melanin-cationic porphyrins complexes. Biophys. Chem..

[55] Saw P.E., Park J., Lee E., Ahn S., Lee J., Kim H., Kim J., Choi M., Farokhzad O.C., Jon S. (2015). Effect of PEG pairing on the efficiency of cancer-targeting liposomes. Theranostics.

[56] Davis M.E., Chen Z.G., Shin D.M. (2008). Nanoparticle therapeutics: An emerging treatment modality for cancer. Nat. Rev. Drug Discov..

[57] Paauwe M., Schoonderwoerd M.J.A., Helderman R., Harryvan T.J., Groenewoud A., van Pelt G.W., Bor R., Hemmer D.M., Versteeg H.H., Snaar-Jagalska B.E. (2018). Endoglin Expression on Cancer-Associated Fibroblasts Regulates Invasion and Stimulates Colorectal Cancer Metastasis. Clin. Cancer Res. Off. J. Am. Assoc. Cancer Res..

[58] Mufamadi M.S., Pillay V., Choonara Y.E., Du Toit L.C., Modi G., Naidoo D., Ndesendo V.M. (2011). A review on composite liposomal technologies for specialized drug delivery. J. Drug Deliv..

[59] Balza E., Castellani P., Zijlstra A., Neri D., Zardi L., Siri A. (2001). Lack of specificity of endoglin expression for tumor blood vessels. Int. J. Cancer. J. Int. Du Cancer.

[60] Rabenhold M., Steiniger F., Fahr A., Kontermann R.E., Ruger R. (2015). Bispecific single-chain diabody-immunoliposomes targeting endoglin (CD105) and fibroblast activation protein (FAP) simultaneously. J. Control. Release Off. J. Control. Release Soc..

