# Supplementary material for: Targeting the Tumor Microenvironment with Fluorescence-Activatable Bispecific Endoglin/Fibroblast Activation Protein Targeting Liposomes
Source: Pharmaceutics. 2020 Apr 17;12(4):370. doi: 10.3390/pharmaceutics12040370 (PMC7238156; doi:10.3390/pharmaceutics12040370)
Supplement: Supplementary file 1 [file pharmaceutics-12-00370-s001.pdf]

Supplementary materials- Pharmaceutics 747169

Targeting the tumor microenvironment with fluorescence-activatable bispecific endoglin / fibroblast activation protein targeting liposomes

Felista L. Tansi[1,*], Ronny Rüger[2,*], Ansgar M Kollmeier[1], Markus Rabenhold[2], Frank Steiniger[3], Roland E. Kontermann[4], Ulf K. Teichgräber[1], Alfred Fahr[2] & Ingrid Hilger[1*]

[1] Dept. of Experimental Radiology, Institute of Diagnostic and Interventional Radiology, Jena University Hospital - Friedrich Schiller University Jena, Am Klinikum 1, 07747 Jena, Germany.

[2] Department of Pharmaceutical Technology, Friedrich-Schiller-University Jena, Lessingstrasse 8, 07743 Jena, Germany

[3] Center for Electron Microscopy, Jena University Hospital - Friedrich Schiller University Jena, Ziegelmuehlenweg 1, 07743 Jena, Germany

[4] Institute of Cell Biology and Immunology, University Stuttgart, Allmandring 31, 70569 Stuttgart

***Correspondence**

felista.tansi@med.uni-jena.de; Tel. +49-3641-9324993 (F. L. T.)

ronny.rueger@web.de; Tel. +49-3641-949905 (R.R.)

ingrid.hilger@med.uni-jena.de; Tel. +49-3641-9325921 (I.H.)

Supplementary data S1: Determination of the melting temperatures of FAP'scFv and mEdg'scFv, their conjugation to micelles and binding of resulting liposomes to target cells.

The single chain antibody fragments (scFv) in form of protein solutions were purified from *E.coli* cultures and the HEK293T cell culture supernatant as reported previously [1,2]. Thereafter, the protein solutions (FAP'scFv and mEdg'scFv) at a concentration of 100–200 µg/ml in PBS were filter sterilized and their melting temperatures determined by dynamic light scattering on a Zetasizer Nano ZS (Malvern, Herrenberg, Germany) by increasing the temperatures in 1 °C steps, with 2 min equilibration duration, starting from 25–70 °C. In these measurements, the melting points of the proteins are characterized by a rash increase in the light scattering intensity measured as evident in the melting curves acquired (Supplementary Figure S1A). Thus, the FAP'scFv and mEdg'scFv revealed melting temperatures in the range 60-63°C and 49-51°C, respectively.

Considering the melting temperatures of the proteins, their binding to the micelles were done at room temperatures whereas the post insertion of the micelles to preformed quenched liposomes was done at 50°C. Thereby, FAP'scFv and mEdg'scFv were conjugated to thioreactive $MalPEG_{2000}$-DSPE and $MalPEG_{3400}$-DSPE micelles respectively, by maleimide coupling. Polyacrylamide gel-electrophoresis (under non-denaturing conditions) of the scFv proteins before (Supplementary Figure S1B, *1*) and after conjugation to the micelles (Supplementary Figure S1B, *2*) revealed characteristic increases in molecular weights which indicated a coupling efficiency of about 90 %. Subsequently, varying molar ratios of the target specific micelles were post-inserted into preformed quenched liposomes to get the resulting targeted-liposome formulations. As can be seen below (Supplementary Figure S1C), using a final 0.1 mol% of the FAP'scFv-specific micelles revealed the most reliable binding to target cells (Supplementary Figure S1C, upper graphic, green line), whereas post- insertion of 0.3 mol% of the mEdg'scFv specific micelles was suitable to produce liposomes with good binding to murine endoglin expressing cells (Supplementary Figure S1C, lower graphic, red line). Thus, bispecific liposomes were generated by post-inserting 0.1 mol% FAP'scFv- and 0.3 mol% mEdg'scFv-targeting micelles.

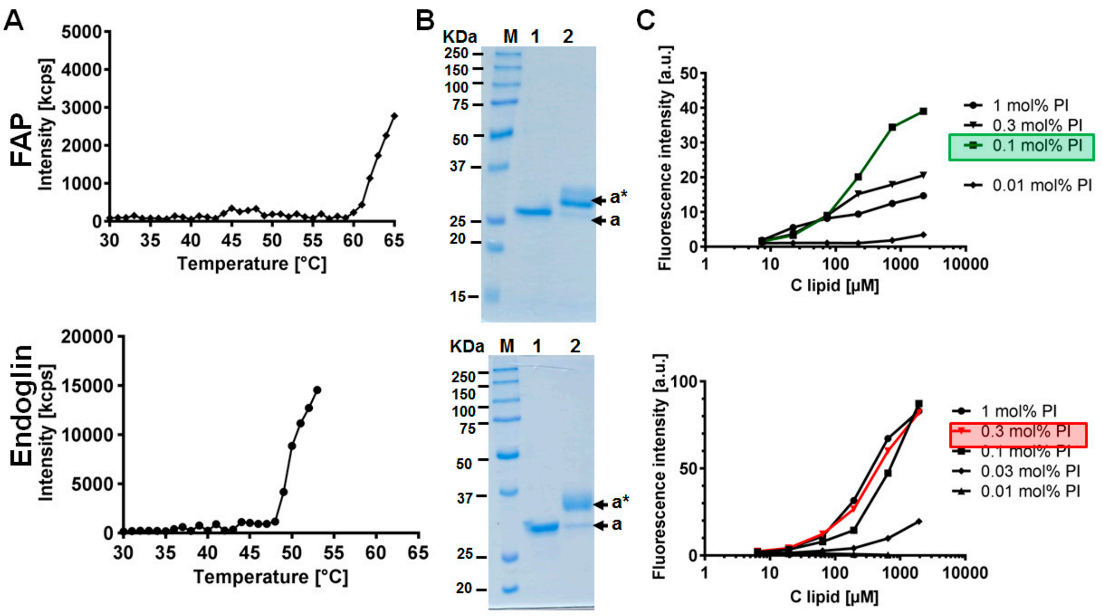

Supplementary Figure S1: A) Representative control analysis of the scFv melting temperature, B) Polyacrylamide gelectrophoresis analysis of the scFv proteins under non-denaturing conditions, before "1" and after conjugation to micelles "2". The letters *a* and *a** depict the free and micelle-conjugated scFv bands, respectively. C) Concentration-dependent binding of targeted liposomes to FAP (upper graph) and murine endoglin (lower graph) expressing target cells, after the insertion of micelles at varying concentrations. Using 0.1 mol% FAP'scFv micelles resulted in the highest binding to the target HT1080-FAP cells (green line), whereas post insertion of 0.3mol% mEdg'scFv micelles showed suitable binding of liposomes to target B16F10-mCD105 cells (red line).

Supplementary data S2: Time course validation of uptake in FAP-expressing cells.

The FAP-positive cell line HT1080-hFAP showed a continuous uptake and activation of the FAP-targeting liposomes (Bi-FAP/mEnd-IL, abbreviated Bis-IL, and FAP-IL) over time after uptake. This is evident in a gradual and persistent increase in the red fluorescence of the near-infrared fluorescent (NIRF) dye, DY-676-COOH encapsulated in the aqueous interior of the liposomes. Thus, the fluorescence could be validated qualitatively by confocal laser scanning microscopy (Supplementary Figure S2A) and semi-quantitatively by NIRF imaging of cell pellets after incubation with the respective liposomes for different durations (Supplementary Figure S2B,C). Whereas the Bi-FAP/End-IL (Bis-IL) is taken up and degraded slowly and is still undergoing activation at 24 h, the FAP-IL is taken up more rapidly by the cells and shows an activation maximum (maximum intensity of DY-676-COOH) already at 12 h (Supplementary Figure S2C). Considering that the Bis-IL carries the mEdg'scFv conjugated on PEG3400 chains, it is likely that this contributes in reducing access of the FAP'scFv to the targets and in turn to the slower uptake and activation.

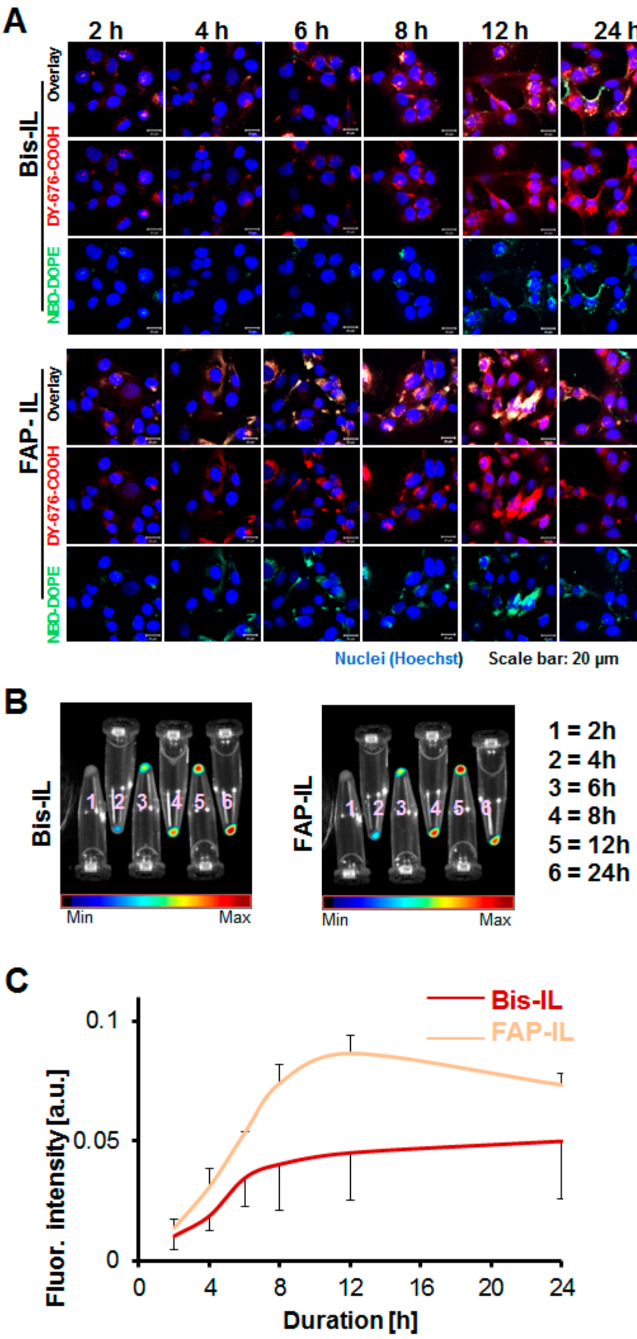

Supplementary Figure S2: A) Confocal microscopic images of HT1080-hFAP cells showing the characteristic fluorescence of liposomal green-fluorescent phospholipid NBD-DOPE and red fluorescence of the encapsulated DY-676-COOH. B). Representative DY 676-COOH based NIRF images of cell pellets overlaid with the white light images after incubation of the cells with liposomes for the indicated durations. C) Corresponding semi-quantitative levels of the DY-676-COOH based fluorescence intensities of the cell pellets. Each line depicts the mean of the fluorescence intensities of 2 independent experiments at the given time points and their standard deviations. No difference (*p>0.05*) could be determined between the mEnd-IL based fluorescence levels at the given time points.

Supplementary data S3: Time course validation of uptake in murine endoglin-expressing cells.

The murine endoglin-positive cell line B16f10mCD105 also revealed a continuous uptake and activation of the murine endoglin-targeting liposomes (Bi-FAP/End-IL, abbreviated Bis-IL, and mEnd-IL) over time after uptake. Thus, a gradual and persistent increase in the liposomal green and red fluorescence was seen. The fluorescence could be validated qualitatively by confocal laser scanning microscopy (Supplementary Figure S3A) and semi-quantitatively by NIRF imaging of cell pellets after incubation with the respective liposomes for different durations (Supplementary Figure S3B,C). Both the Bis-IL and mEnd-IL were taken up and degraded rapidly, and exposed an activation maximum (maximum intensity of DY-676-COOH) already at 12 h and 8 h respectively (Supplementary Figure S3C). Therefore, the fact that mEdg'scFv was conjugated to PEG_{3400} chains seems to be advantageous for Bis-IL-based binding to murine endoglin on the cell surface with no visible influence caused by the FAP'scFv.

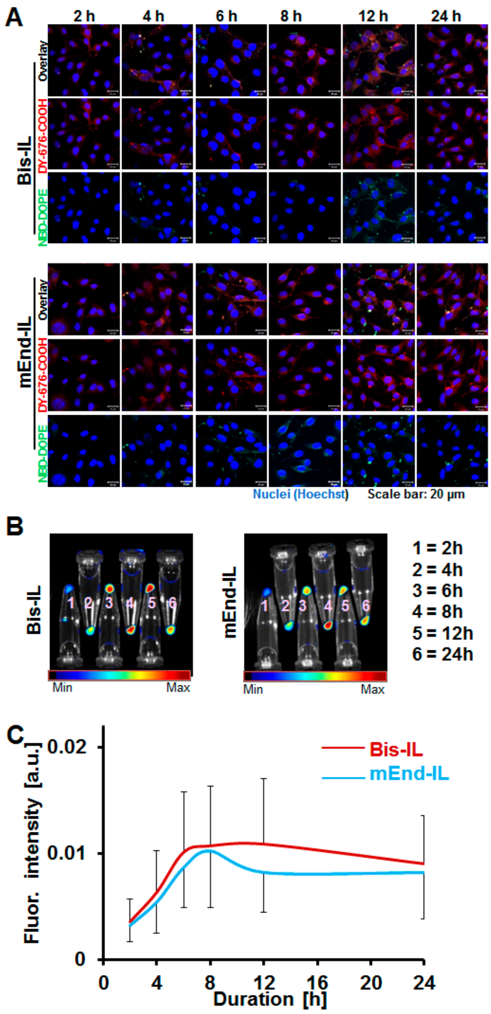

Supplementary Figure S3: A) Confocal microscopic images of B16F10mCD105 cells showing the characteristic fluorescence of the liposomal green-fluorescent phospholipid NBD-DOPE, and the red fluorescence of the

encapsulated DY-676-COOH. B). Representative DY-676-COOH based NIRF images of cell pellets overlaid with the white light images after incubation of the cells with liposomes for the indicated durations. C) Corresponding semi-quantitative levels of the DY-676-COOH based fluorescence intensities of the cell pellets. Each line depicts the mean of the fluorescence intensities of 2 independent experiments at the given time points and their standard deviations. No difference ($p>0.05$) could be determined between the mEnd-IL based fluorescence levels at the given time points.

Supplementary data S4: Tumor to background ratios

In vivo NIRF imaging of HT1080-hFAP based xenografts which express high FAP levels on the tumor cells, tumor associated fibroblasts and also murine endoglin on the tumor vasculature revealed gradual and increasing tumor-to-background ratios (TBR) for all the liposomes (Supplementary Figure S4). Thereby, the FAP-targeted liposomes Bis-IL and FAP-IL resulted in ratios higher than3 as from 4 h post intravenous injection and increased gradually over time reaching TBRs of almost 6 at 48 h. Contrary to these, the mEnd-IL showed TBRs of approximately 3 only as from 30 h p.i. when the background is reduced, indicating a long retention of the mEnd-IL based fluorescent components within cells after uptake and activation. In addition, the control LipQ accumulated in the tumors comparably rapidly and yielded TBRs higher than 3 as from 4 h p.i., but remained below 5 throughout the investigation time (Supplementary Figure S4, upper panel).

In contrast to the HT1080-hFAP based xenografts, the target-negative MDA-MB231-based xenografts which should express low FAP levels on the tumor associated fibroblasts and also murine endoglin on the tumor vasculature revealed much lower but gradually increasing TBRs for all the liposomes. These approached a value of 3 as from 24 h p.i. for the FAP-targeted liposomes Bis-IL and FAP-IL and the control LipQ, and remained below 4 for all the liposomes throughout the investigation time (Supplementary Figure S4, lower panel). TBRs for the mEnd-IL were 3 only as from 26 h post injection and remained unchanged till the end of the observation.

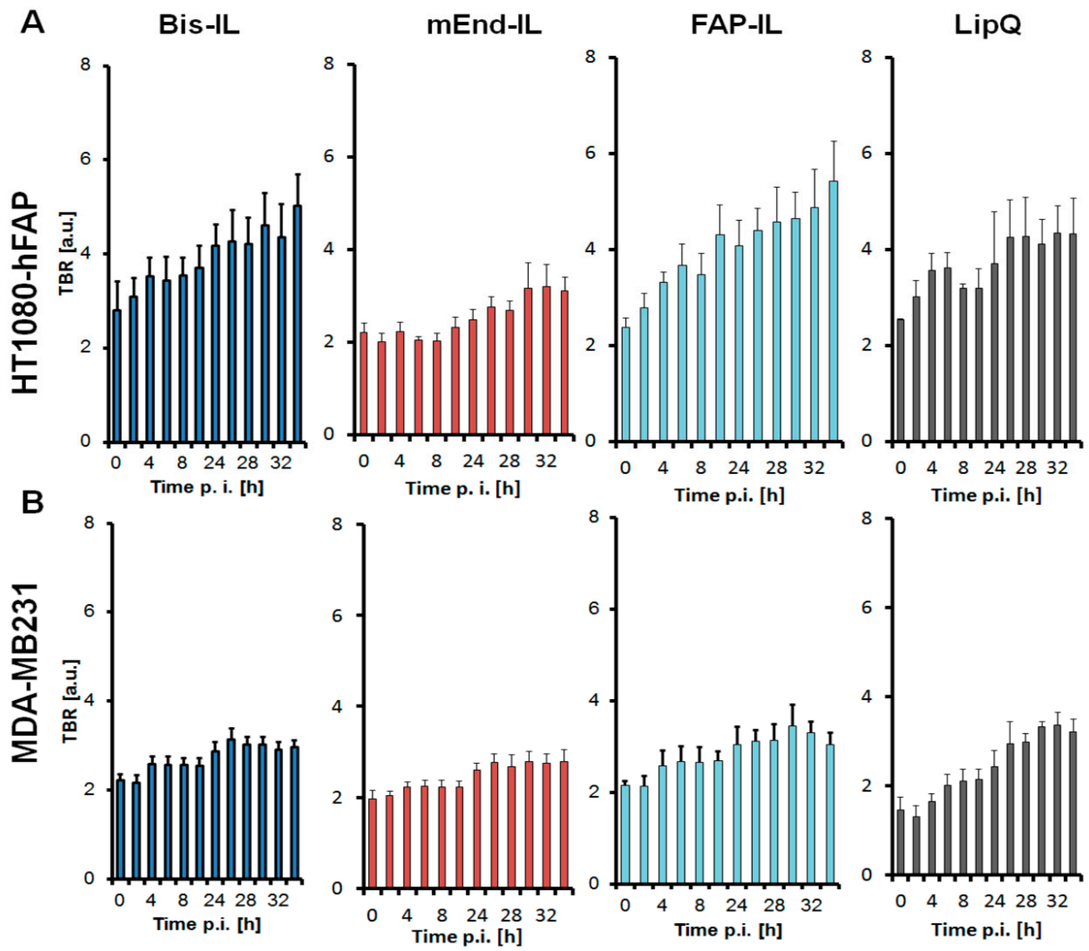

Supplementary Figure S4: Tumor to background ratios of the fluorescence intensities detected in the high FAP-expressing HT1080-hFAP and the target negative MDA-MB231 based xenografts. For the HT1080-hFAP, each bar represents the mean of n = 7 (Bis-IL), n = 3 (mEnd-IL), n = 5 (FAP-IL), n = 5 (LipQ), and the respective SEM. For the MDA-MB231, each bar represents the mean of n = 7 (Bis-IL), n = 7 (mEnd-IL), n = 5 (FAP-IL), n = 6 (LipQ), and the respective SEM.

References

1. Rüger, R.; Müller, D.; Fahr, A.; Kontermann, R.E. Generation of immunoliposomes using recombinant single-chain Fv fragments bound to Ni-NTA-liposomes. *Journal of drug targeting* **2005**, *13*, 399-406, doi:10.1080/10611860500353328.
2. Rüger, R.; Tansi, F.L.; Rabenhold, M.; Steiniger, F.; Kontermann, R.E.; Fahr, A.; Hilger, I. In vivo near-infrared fluorescence imaging of FAP-expressing tumors with activatable FAP-targeted, single-chain Fv-immunoliposomes. *Journal of controlled release : official journal of the Controlled Release Society* **2014**, *186C*, 1-10, doi:10.1016/j.jconrel.2014.04.050.
